# Synthesis, physico-chemical characterization, and environmental applications of meso porous crosslinked poly (azomethine-sulfone)s

**DOI:** 10.1038/s41598-022-17042-0

**Published:** 2022-07-27

**Authors:** Marwa M. Sayed, Mohamed Abdel-Hakim, Mahmoud H. Mahross, Kamal I. Aly

**Affiliations:** 1 Chemistry Department, Faculty of Science, New Valley University, El- Kharga, 72511 Egypt; 2 Chemistry Department, Faculty of Science, Al-Azhar University, Assiut, 71524 Egypt; 3 Polymer Laboratory 122, Chemistry Department, Faculty of Science, Assiut University, Assiut, 71516 Egypt

**Keywords:** Organic chemistry, Polymer chemistry, Materials science

## Abstract

To develop innovative mesoporous crosslinked poly(azomethine- sulfone)s with environmental applications, a simple Schiff base condensation technique based on barbituric acid BA or condensed terephthaldehyde barbituric acid TBA in their structures as monomeric units are applied. Different analysis methodologies and viscosity measurements identify them as having stronger heat stability and an amorphous structure. The photophysical features of the multi stimuli response MSR phenomenon are observable, with white light emission at higher concentrations and blue light emission at lower concentrations. Their emission characteristics make them an excellent metal ions sensor through diverse charge transfer methods. They can have a better inhibition efficiency and be employed as both mixed-type and active corrosion inhibitors according to their fluorescence emission with metals, demonstrating their capacity to bind with diverse metals. The adsorption of two distinct dye molecules, Methylene blue MB cationic and sunset yellow SY anionic, on the mesoporous structures of the polymers is investigated, revealing their selectivity for MB dye adsorption. Quantum studies support these amazing discoveries, demonstrating a crab-like monomeric unit structure for the one that is heavily crosslinked.

## Introduction

Crosslinked network polymers have amazing thermal, mechanical, and chemical stability properties and can be transformed into reusable materials using crosslinked bonds redistributing around the network^1^. This crosslinking can be accomplished in two ways: via a supramolecular reaction^2–4^ or by forming a dynamic covalent link^5,6^. Covalent Adaptable Networks are a new type of dynamic polymer (CAN) and crosslinked materials that are recyclable while retaining thermo-solid plastic under particular stimuli. As a result, they have both thermoset and thermoplastic characteristics. Reversible Diels–Alder (DA)^7–9^, hydrazone, oxime, transesterification, and polyimine are among the CANs^10^.

Polyimine (PI), polyazomethine, or Schiff base polymers are accessible as they can be made by combining various aliphatic or aromatic diamine types with dialdehyde or diketone^11–13^. The PI has gotten much attention because of its remarkable thermal stability, strength, and modulus^14^ high-performance fibers and film-forming polymers, semiconductor materials^15^, nonlinear optical characteristics^16^, and the capacity to produce chelates are becoming increasingly attractive. However, many polymers can also be heated or dissolved to generate intermediate phases^17^, and others have high melting temperatures and limited solubility, complicating their characteristics and processing. The first step toward creating a processable PI is to provide flexible bonding between the aromatic rings, such as (–O–, –CH_2_–S, –SO_2_– etc.) that results in asymmetrical monomeric units on a skeleton^18,19^. Among the PI's remarkable qualities are its great capacity to coordinate and attach to metal ions via the imine link, Bronsted acid, or Lweis acid. Aldehyde, ketone, and amine structures must be modified to provide new functions, increase chelate stability, and have other unique characteristics of large molecules (–OH, –CN, –NH, –NO_2_, –SO_2_, etc.)^20^. Barbituric acid BA has a unique polyfunctional structure and is thermally stable due to the presence of two amine donors and three carbonyls^21^. These amino groups can encourage supramolecular assembly through hydrogen bonding and create a polymer structure by metal bonding^22,23^.

Adding a sulfone group to the PI structure should result in novel materials with improved characteristics and a striking balance between the isotropic sulfone group and the rigid anisotropic imine bond, which are known as poly(azomethine-sulfone) (PAS) or poly(imine-sulfone) (PIS)^24,25^. Polysulfone (PSF) is a translucent engineering plastic with a sulfone group in its backbone commonly used in medical and food processing equipment, electrical and electronic components, camera boxes, and pipes^26,27^. These groups can chelate with transition metal ions in their solution, changing the nature of their optical characteristics and potentially serving as a useful sensor for these metals^28–30^. Corrosion of metals and steel, used in every industrial sector as medical, construction, and transportation means, is one of the problems that can harm the economic, engineering, and scientific worlds. Scientists are working to solve this problem by finding simple, safe, and inexpensive ways to prevent this phenomenon^31^. Inhibitors, which contain a heteroatom, electron-rich group, or conjugated system, are the most efficient strategy to avoid corrosion; Schiff base and polymers are in the middle^32–36^.

Another issue resulting from industrial and agricultural waste is the discharge of colored contamination into the aquatic ecosystem, containing hazardous organic or inorganic chemicals such as dyes^37^. Several methods are applied to remove these substances, like adsorption, ozonation, coagulation, and flocculation. Removing dyes from water systems has been a technical challenge for decades^38^.

The major objective of this work is to modify and enhance the fascinating features of poly(azomethine-sulfone) from chelation and porosity by crosslinking technique, which will be mediated through hydrogen bonding of the baribituric acid moiety. Two monomeric components, barbituric acid (BA) and condensed terephthaldehyde barbituric acid (TBA), combine with diamino sulfone via a Schiff base reaction to form two distinctive outstanding network poly(azomethine-sulfones). Different methods are used to chemically and physically characterize the produced network polymers. They exhibit intriguing properties for various uses, including metal sensors, corrosion inhibitors, and rapid dye adsorption from aqueous solutions.

## Experimental

### Materials

Barbituric acid, terephthaldehyde (Alfa Aesar), 4,4^`^-diamino diphenyl sulphoxide (El Nasser chemicals), tetrahydrofuran (THF), dimethyl sulfoxide (DMSO), zinc acetate ((CH_3_COO)_2_Zn), ferric (III) chloride anhydrous (FeCl_3_), copper (II) acetate hydrate ((CH_3_COO)_2_Cu), hexahydrate cobalt (II) chloride (CoCl_2_) and nickel (II) sulfate (NiSO_4_), sulfuric acid H_2_SO_4_, methanol, dimethylformamide DMF, are obtained from Sigma-Aldrich, methylene blue dye, colorant sunset yellow dye (Al Gomhouria Co.) and used without purification.

### Measurements

(FT-IR) infrared spectroscopies are measured using Shimadzu 2110 PC scanning spectrometers by the KBr method. Raman spectroscopy is performed on a Nicolet IS50 spectrometer (Thermo Scientific, USA) to study the chemistry of the bonds. JEOL (ECA 500) spectrometer is used for getting nuclear magnetic resonance (^1^H-NMR) and (^13^C NMR) spectra by using deuterated DMSO-d_6_ as a solvent. X-ray diffraction crystallography (XRD) is performed using powdered samples technique to study the crystalline nature them via scan type Coupled two theta/theta (2θ) and PSD fast mode continuous with detector SSD160 (1D model). Thermal Analyzer TA Q-600 measures thermogravimetric analysis (TGA) and Differential scanning calorimetry (DSC) with a heating rate of 10 °C/min under an N_2_ atmosphere. UV–Vis spectra are measured on Shimadzu mini 1240. The fluorescence emission spectra are done at room temperature by Hitachi F-7100 FL Spectrophotometer. Recognition of the surface morphology of the designated polymers by Jeol JSM-5400 LV scanning electron microscope (SEM) device via coating method. Gel permeation chromatography (GPC) (PL-GPC-220, high-temperature chromatography, Agilent Technologies, USA) was used to measure the molecular weight in Dimethyl formamide (DMF), with a flow rate: of 1.0 mL/min. Quantum chemical calculations are made with the Gaussian 09^39^ in the gaseous phase employing the density functional theory method with the B3LYP functional ^40^ and the 6-311++G (d,p) basis set. Electrochemical studies (linear, Tafel plots polarizations) and open circuit potential tests utilize 352/252 model corrosion measurement procedures. This method includes an EG & G potentiostat/galvanostat model 273A that employs software from IBM.

### Synthesis of terephthaldehyde bis barbituric acid (TBA)^41,42^

In two necked flask, 250 ml of about (3.82 mg, 2 mmol) of barbituric acid (BA) with (2 mg, 1 mmol) of terephthaldehyde (TPh) are heated under reflux with stirring in 70 ml absolute ethanol for about half an hour, until all substances are completely dissolved, and a clear solution is obtained. Then 2–3 drops of piperidine are added as a catalyst; a yellow precipitate is obtained by continuous refluxing the reaction for about two hours. After that, the reaction mixture is cooled gradually, the solid precipitate is filtered from the solution, rinsed several times with ethanol, dried in an oven at 70 °C, and hot ethanol is used for purification to afford a canary yellow powder in a good yield (4.8 mg, 93%), m.p. > 350 °C. ^1^H NMR (500 MHz, DMSO-d_6_) δ 11.62 (s, 2H_a_), 11.21 (s, 2H_b_), 8.19 (s, 2H_c_), 8.10–7.07 (m, Ar–H, C–H aromatic). ^13^C NMR (126 MHz, DMSO-d_6_) δ 162, 153.26, 150.70, 132.32, 128.81, 120,08.

### Preparation of poly(azomethine-sulfone) (PSF1)

Polymer PSF1 is prepared by dissolving approximately (1 mmol, 128 mg) of barbituric acid (BA) with (3 mmol, 745 mg) of 4,4`-diamino diphenyl sulfone (Dapsone) in about 25 ml DMSO as a solvent in a round flask 100 ml under N_2_, after degassing the mixture is refluxed at 180 °C for about 72 h by a heating mantel. After cooling, the polymer is precipitated with water, filtered, washed several times by THF, and dried in an oven at 70 °C for 24 h to afford a yellow powder with a yield (82%). ^1^H NMR (500 MHz, DMSO-D6) δ 8.51–6.5 broad (Ar–H), 4.51–5.5 (NH), 3.21 (CH_2_).

### Preparation of poly(azomethine-sulfone) (PSF2)

The network polymer PSF2 is prepared similarly to (PSF1). In a round flask of 100 ml under N_2_ atmosphere, about (1 mmol, 354 mg) of TBA with (3 mmol, 745 mg) of 4,4`-diamino diphenyl sulfone (Dapsone) are dissolved in about 30 ml DMSO as a solvent. The reflux is also continued at 180 °C for about 72 h on a heating mantel. The polymer is gotten after cooling by precipitating with water and filtered. PSF2 is washed several times by THF and then dried in an oven at 70 °C for 24 h to afford a faint yellow powder with a yield (of 52%). ^1^H NMR (500 MHz, DMSO-D6) δ 8.80–6.30 (Ar–H), 3.6–4.7 (NH).

### Preparation of the corrosive media, test specimens, and inhibitor solutions

The corrosive medium in this test is (1.0 M H_2_SO_4_) and is prepared from annular grade H2SO4. All examinations are performed in the air. The test accomplishment of the mild steel specimens is done at the Tabbin Institute for Metallurgical Studies in Cairo. The patterns consist mainly of 98.74% Fe with few percent of C 0.17%, Mn 0.71%, Si 0.022%, Cr 0.045%, P 0.010%, Al 0.0017%, Ni 0.072%, Cu 0.182%, F 0.022%, Mo 0.011% and Sn 0.011%. To achieve a smooth surface on the specimen, 1000–1400 grade emery paper is used to refine the electrode's face, distilled water is used to rinse, and acetone is used to degrease. After ten minutes, the electrode is rinsed with water and dried with filter paper^43^. Inhibitor's solution of PSF1 and PSF2 are prepared with a concentration of 500 ppm in DMSO. The technique used for experiments is coating by forming a thin film on the mild steel electrode with a thickness of 4 µm (measured by a micrometer caliper). It is immersed in the corrosive medium to begin the potentiodynamic behavior.

## Results and discussion

**Figure 1:**
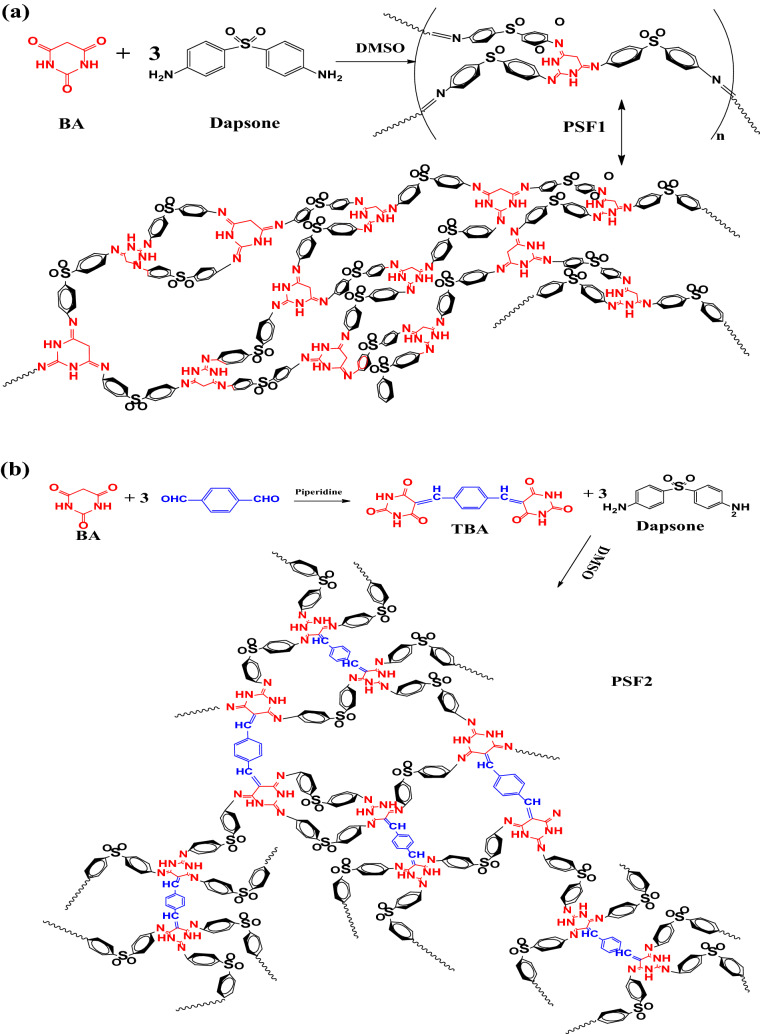
(**a**) Preparation of PSF1. (**b**) Preparation of TBA and PSF2.

**Figure 2:**
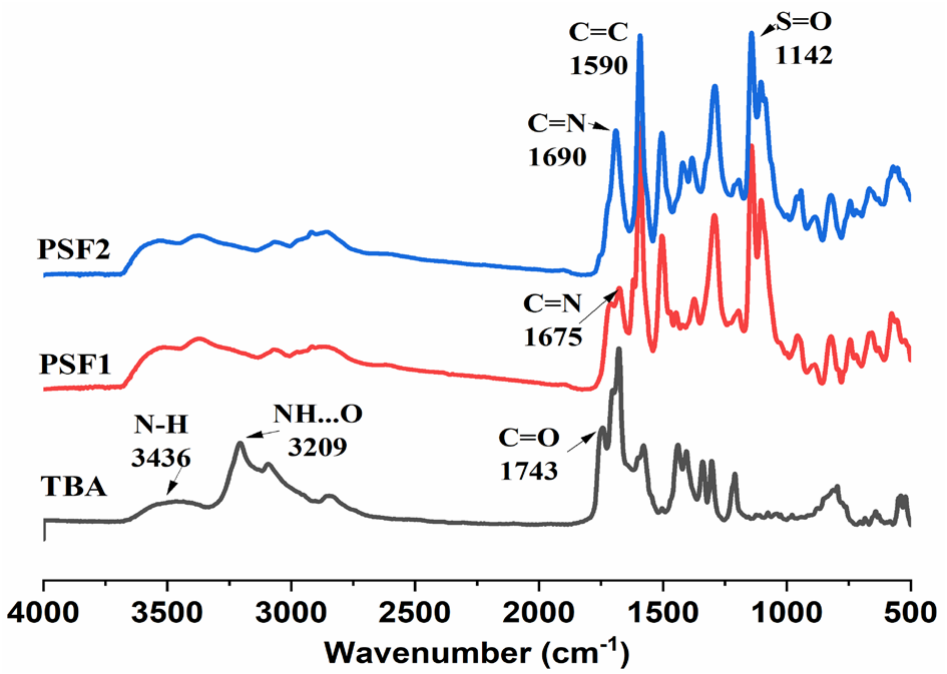
FT-IR of TBA, PSF1, and PSF2.

As indicated in Fig. 1, the network polymers are made by Schiff base condensation under the influence of heat. PSF1 is made by reacting BA in DMSO with three equivalents of Dapsone for 72 h. Whereas PSF2 is made in the same way from 1 equivalent, TBA condensed monomeric unit of BA with 3 mol equivalents of Dapsone. BA is a heterocyclic pyrimidine molecule with an active methylene group that can yield C=C from aldehydic derivatives via a simple condensation reaction. In the presence of piperidine as a basic catalyst, terephthaldehyde is employed to make TBA in an ethanolic solution with 2 equivalents BA. Due to the unique ability of BA as a self-assembling molecule through hydrogen bonding, the variation of these building units leads to the formation of diverse polymers with different surface areas and properties^22,44^. ^1^H-NMR, ^13^C-NMR, and FT-IR emphasize the structure of TBA. As displayed in the ^1^H-NMR spectrum in Fig. S1, the characteristic bands of TBA in (DMSO-d_6_) are found at 11.62, 11.21 ppm for NH, 8.19 ppm for CH=C, and 8.10–7.07 ppm for the aromatic ring. Figure S2 represents the resonance of carbon atoms for carbonyl groups adjacent to (CH=C–C=O) at 162 ppm, adjacent to (NH–C=O) at 150.70 ppm, at 153.26 ppm for pyrimidine carbon that condensed with Tph (C=CH), 132.32 and 128.81 ppm are for aromatic carbons, 120.08 ppm for condensed aldehyde atom. Figures S3,4 show ^1^H-NMR of PSF1 and PSF2 in (DMSO-d_6_), respectively. The bands are broad due to the polymeric nature and appearance of NH bond in the range of about 4.51–5.5 ppm for PSF1 and at 3.6–4.7 ppm for PSF2; this is attributed to hydrogen bonding alongside the increase of electron density around the H atom of NH as calculated from DFT calculations^45^. Figure 2 exhibits FT-IR spectra of TBA monomer and polymers, the spectrum of TBA demonstrates peaks at 3436, 1743, 1678 cm^−1^ equivalent to N–H stretching bond, C=O stretching vibration, and CH=C resulting from condensation reaction^46^; there is a peak at 3209 cm^−1^ for hydrogen bonding between NH and nearing O atom of C=O^47^. The resulting polymers' spectra show the carbonyl group's disappearance and the occurrence of imine bond C=N at 1675 cm^−1^ for PSF1 and also at 1690 cm^−1^ for PSF2. Besides that, peaks of N–H are shifted to 3369 cm^−1^, and the presence of another peak at 3550 cm^−1^ may be due to intramolecular or extended hydrogen bonding.

Furthermore, the peaks at 1590 cm^−1^ and 1142 cm^−1^ represent alkene C=C and sulfone groups, respectively. The Raman spectrum can be a tool for studying the structure of solid polymers, especially the orientation order of large molecules. Figure S5 displays the Raman spectra of polymers; the spectra contain peaks at 782 and 3510 cm^−1^ corresponding to asymmetric C–S–C bond and N–H bond, respectively. The peaks show the isotropic molecular orientation of these polymers^48,49^.

### Computational studies

**Figure 3:**
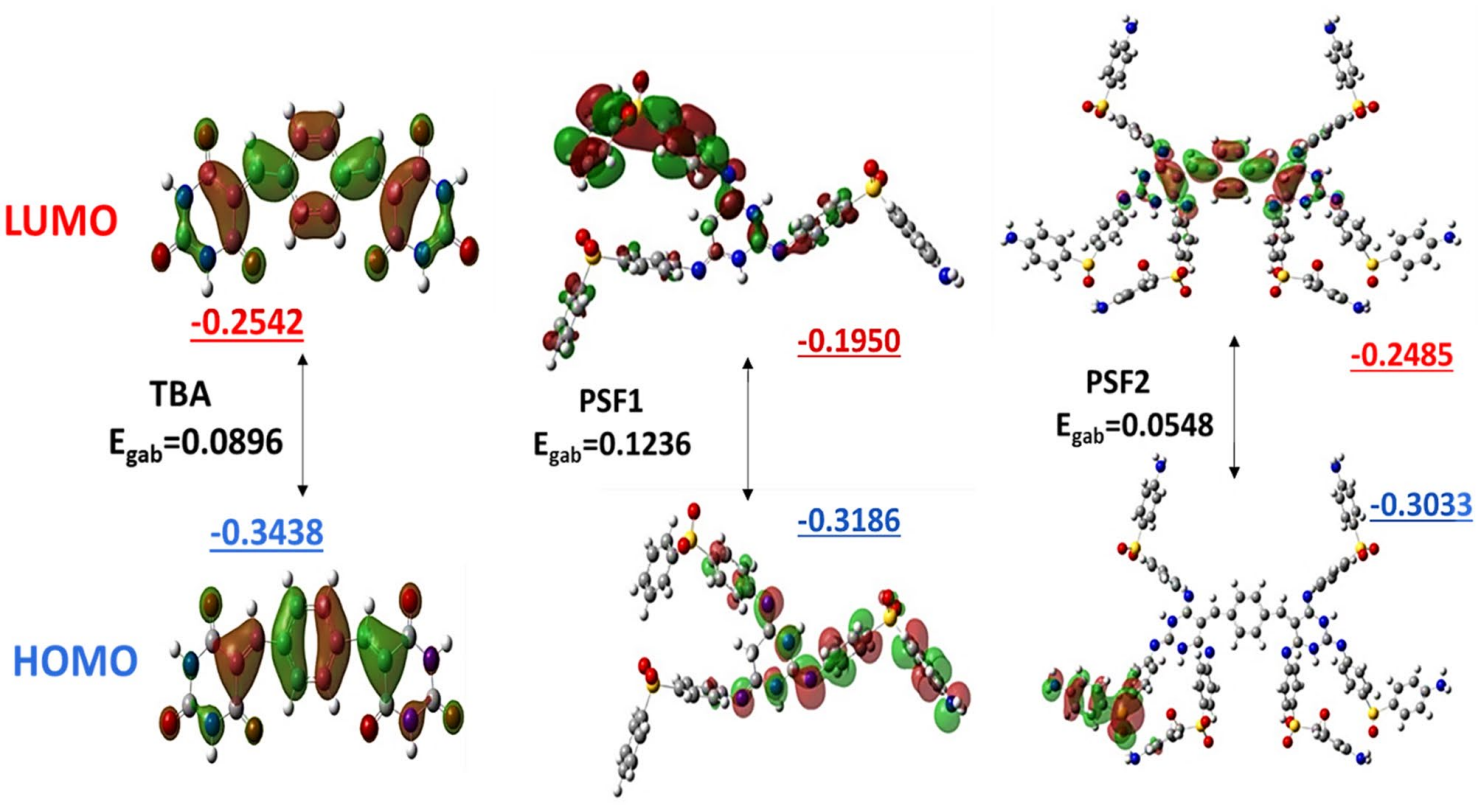
The shapes of HOMO, LUMO of TBA, PSF1, and PSF2 were calculated by using DFT, B3LYP/6-31G (d,p).

**Figure 4:**
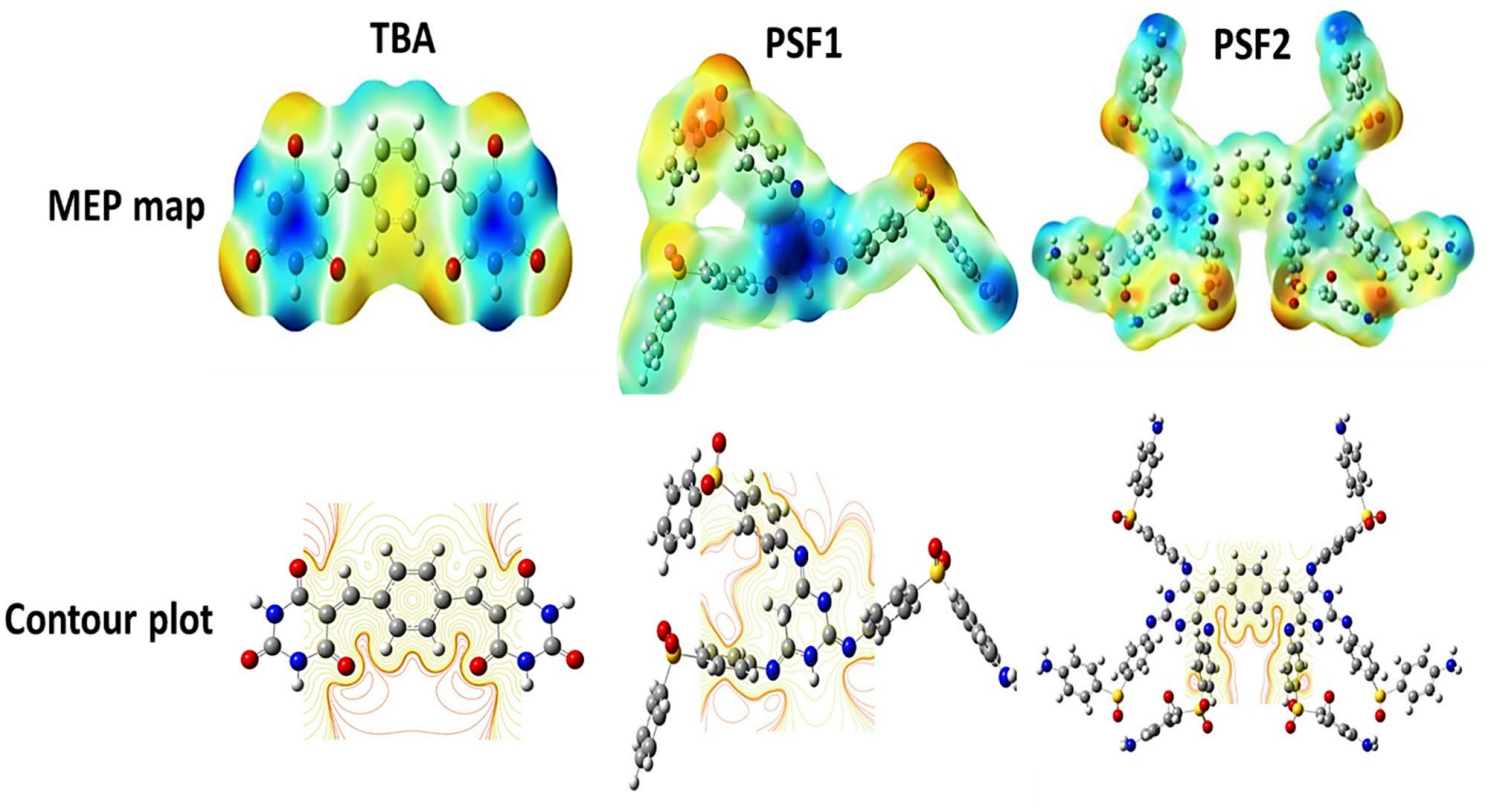
MEP and Contour of TBA, PSF1, and PSF2 were calculated using DFT, B3LYP/6-31G(d,p). Atom color code: C (gray), H (white), O (red), N (blue) and S (yellow).

An overall process for detecting the configuration and degree of electronic charge in the molecule is the Density functional theory (DFT). Quantum chemical computations are performed on representative monomeric units of TBA, PSF1, and PSF2. Geometry optimization is used to find the forced structural changes in a molecule or the arrangement of molecules in space where the forces between the net atoms are near zero^50^. The shape of the monomeric units is depicted in Fig. S6 using DFT, B3LYP/6-31G. (d, p). It displays that BA moieties in the TBA molecule exist in a transform related to the π bond that bonds the benzene ring that acts as a donor and BA as an acceptor^51^. The monomeric units of polymers contain the Dapsone molecule, which has an envelope shape, great stability, and heavy electron density on its oxygen atoms. This density makes this region more electrophilic and tends to form intermolecular hydrogen bonding between molecules^52^. The geometry results show that the PSF2 monomeric unit is shaped like a crab. Table S1 represents the lowest unoccupied (LUMO), highest occupied (HOMO) molecular orbital energy and energy gap between the monomeric units. It is found from these results that the TBA molecule has a lower energy gap than the monomeric unit of PSF1, and this appears clear in the PSF2; when this molecule TBA becomes a structural unit in its molecular structure PSF2, the lowest value of the energy gap is obtained. Their shapes are displayed in Fig. 3. Estimating the reactivity of molecules towards positive or negative charge reactants is usually through the electrostatic potential of molecules. Their values can be imagined by drawing onto the molecule's surface identifying the borders of the molecules; molecular electrostatic potential (MEP) can be used for this. Figure 4 displays MEP and contour plots of molecules. The red color indicates that the region is highly attractive, and the blue color shows the repulsion in this region. The more negative areas of these molecules are concentrated on the sulfone group in the matrix, and repulsive regions exist in regions containing BA moiety. The repulsion in this area is produced by the nitrogen atoms' non-bonding electrons, causing the NH bond's electron density to grow^45^.

### Physico-chemical characterization

#### Solubility and viscosity

The network polymers PSF1 and PSF2 are soluble in DMSO, DMF, N-methyl pyrrolidone, and concentrated sulfuric acid. They are not soluble in chloroform, methylene chloride, and THF. Their solubility in polar solvents may be due to the NH bond in these molecules. The number of crosslinks between junctions, the distribution of monomer units along the polymer backbone, and chain branching all have an impact on how soluble polymers are. It is the summation of the contributions of hydrogen bonding, polar forces, and dispersion forces. If the crosslinked polymers exhibit maximal equilibrium swelling in the solvent, it is assumed that the solubility parameter of the crosslinked polymer is similar to that of the solvent. The resultant polymers dissolve in DMSO and DMF may be due to the similarity in their solubility parameters that leads to more swelling of polymers in these solvents and solvation^53^. The inherent viscosity $\eta_{inh}$ of these polymers is measured in DMSO and calculated from the equation:

$$\eta_{inh} = \frac{{2.3\log \frac{\eta }{{\eta_{o} }}}}{{\text{C}}}$$

where: ${\eta }_{o}$ and $\eta$ : the viscometer movement time for the solvent (DMSO) and the tested polymers, respectively, C: sample concentration (0.05 g/100 mL). The results are 2.16, 1.33 dl/g for PSF2, PSF1. The higher value is for PSF2, which may be due to the higher branching and aggregation of layers leading to slow solution movement^54^. A GPC approach was used to calculate the synthesized PSF's molecular weight and polydispersity index (PDI), by dissolving 4 mg of sample in DMF at a temperature of 50 °C and employing polystyrene standards and the universal calibration mode; the refractive index detector was calibrated. The results are illustrated in Fig. S7 and Table S2, indicating the higher molecular weight of the resulting polymers PSF1 has 904 repeating units, and PSF2 has 511 repeating units. This difference in results from Viscosity measurements may be due to the drawbacks of "traditional" GPC, which determines molecular weights by comparing them to specific polymer standards. The molecular weights are only reliable if the investigated polymer has a similar chemistry to the stanadard. The analysis of branched polymers also introduces additional error because, when branching is introduced into a polymer structure, the molecular weight and hydrodynamic size do not rise correspondingly. As a result, viscosity measurements via universal calibration were also used to determine the molecular weights. Compared to traditional GPC, this makes it possible to acquire significantly more precise molecular weights^55^.

### Thermal stability

**Figure 5:**
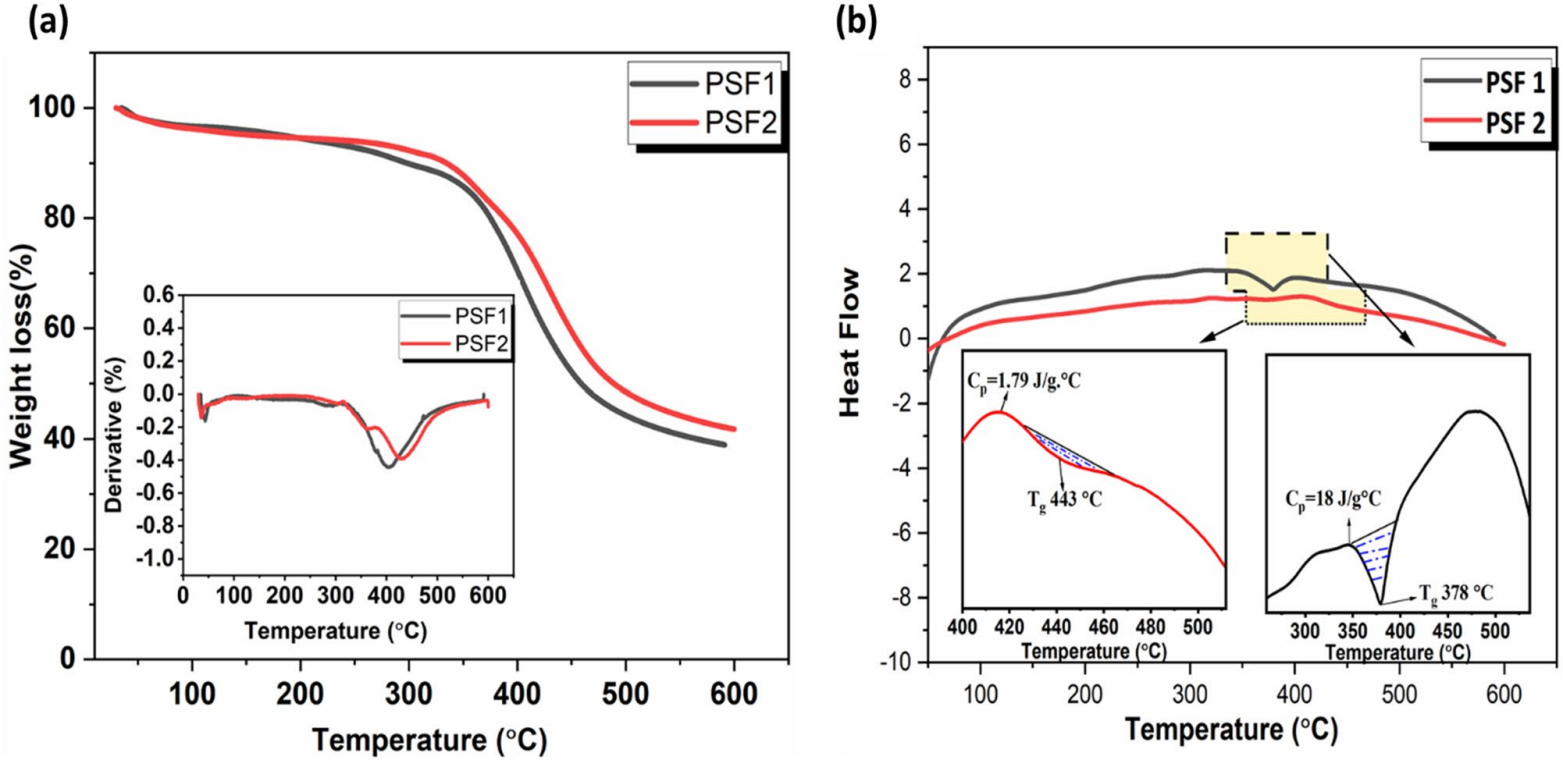
(**a**) TGA and Dr-TGA inset of PSF1 and PSF2 (**b**) DSC of PSF1 and PSF2.

The persistence of the prepared polymers against heat is measured by thermogravimetric analysis (TGA) and differential scanning calorimetry (DSC) techniques. Figure 5a,b and Table 1 display the gravimetry degradation behavior of these polymers and their states; it appears clearly from derivative loss curves that there are three stages of weight loss in all samples. The first loss is due to the evaporation of solvents adsorbed on the surface of these polymers, the second and third are attributed to the degradation of samples. The start decomposition of polymer is at 10% weight loss; the temperatures of 10% weight loss are 298 and 334 °C for PSF1, and PSF2, respectively. The higher char yield at 600 °C is 42% for PSF2. The thermograms of DSC Fig. 5b have been recorded during a heating cycle at a heating rate of 10 °C min^−1^. The two polymers show only a trace inflection corresponding to glass transition (Tg). Curves of DSC show that (T_g_) are 378, 417 °C for PSF1 and PSF2, respectively. These values denote the higher crosslinking density in these polymers, which leads to restriction in the movement of their molecules and record a higher value in PSF2 than PSF1^56–58^. Correspondingly, the specific heat capacities C_p_ of PSF1 and PSF2 at the transition start are 18 and 1.79 J/g °C. The lower value of heat capacity of PSF2 than PSF1 elucidates that the atoms are more closely together and can transfer heat easily from one atom, exciting the other atoms next to it^59^. These results deduce that PSF2 is more rigid and highly branched and has the highest thermal stability.

**Table 1:** Temperature (°C) for various decomposition levels in N_2_ at a heating rate of 10 °C/min.

Polymer	10% wt. Loss	20% wt. Loss	30% wt. Loss	40% wt. Loss	50% wt. Loss	Char yield (%) at 600 °C
PSF1	298	375	401	424	458	39
PSF2	334	388	421	448	488	42

### X-ray analysis and surface morphology

**Figure 6:**
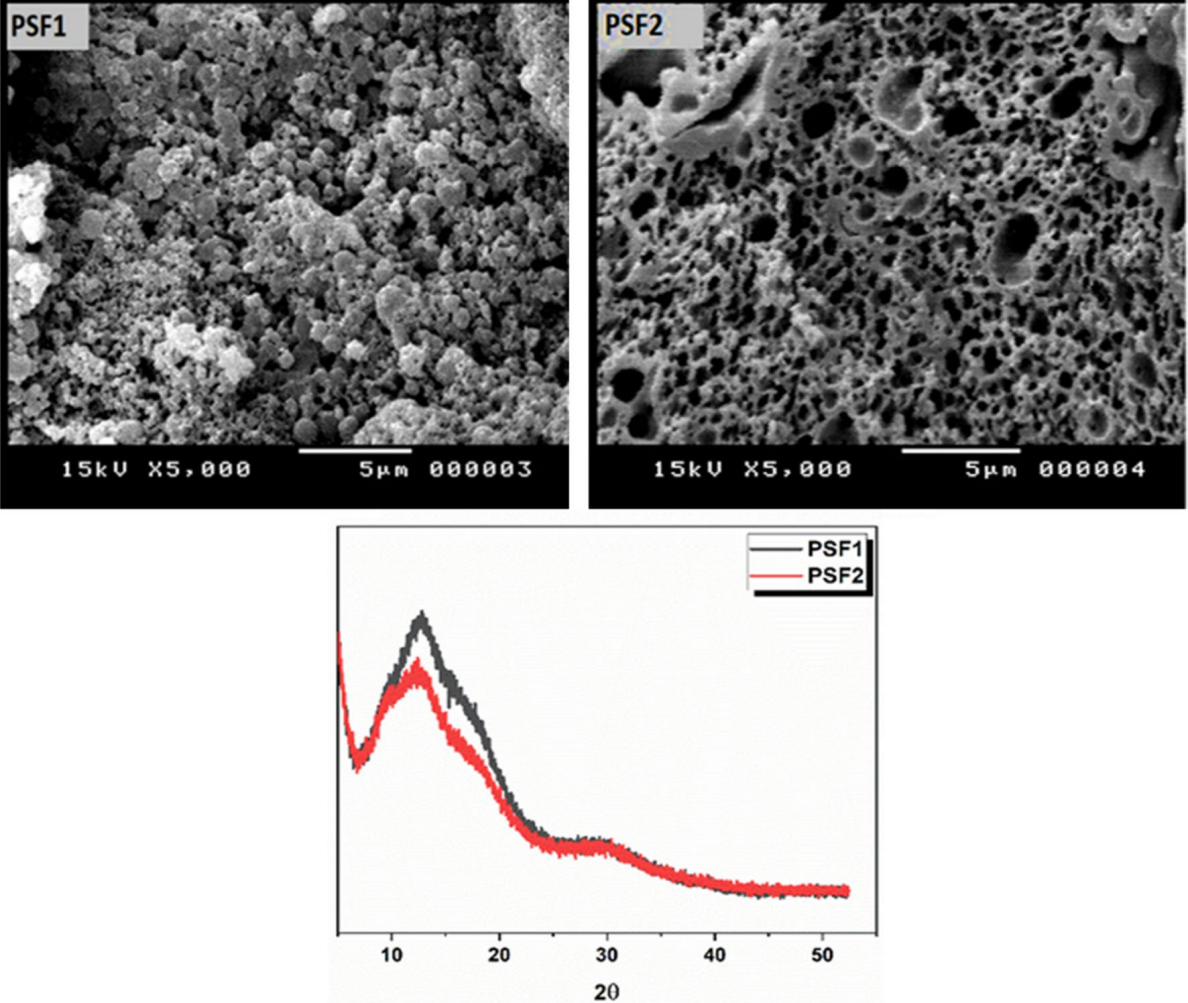
X-RD and SEM images at magnification (X = 5000) of PSF1, PSF2.

The amorphous or crystalline structure of a polymer can be detected through the X-ray diffraction technique. When the incident beam of the x-ray interferes with the sample plane, the diffraction pattern is produced, indicating sample uniformity. Figure 6 exhibits the diffraction of prepared polymers PSF1and PSF2; the crystallinity percent of PSF1 is about 34.9% and lower in broadening of the peak with an amorphous percent of 65.1%. PSF2 has a crystallinity percent of about 27.1% and an amorphous portion of 79.9%, with a broader diffraction peak than the first one. These data indicate their amorphous structures and higher folding and assembling of the chains due to hydrogen bonding and network structure, which appear clearly in PSF2. The microscopic morphology of polymers PSF1 and PSF2 is demonstrated in Fig. 6 with magnification (X = 5000). SEM image of PSF1 has in closed packed and rough spherical structure with a diameter of 5 µm. In comparison, PSF2 at the same magnification has net or voids and a porous honeycomb structure with pores < 5 µm. the increase in crosslinking inside the molecule's structure leads to more packing and porous features in the resulting polymer^60^.

### Photophysical properties and metals sensitivity

**Figure 7:**
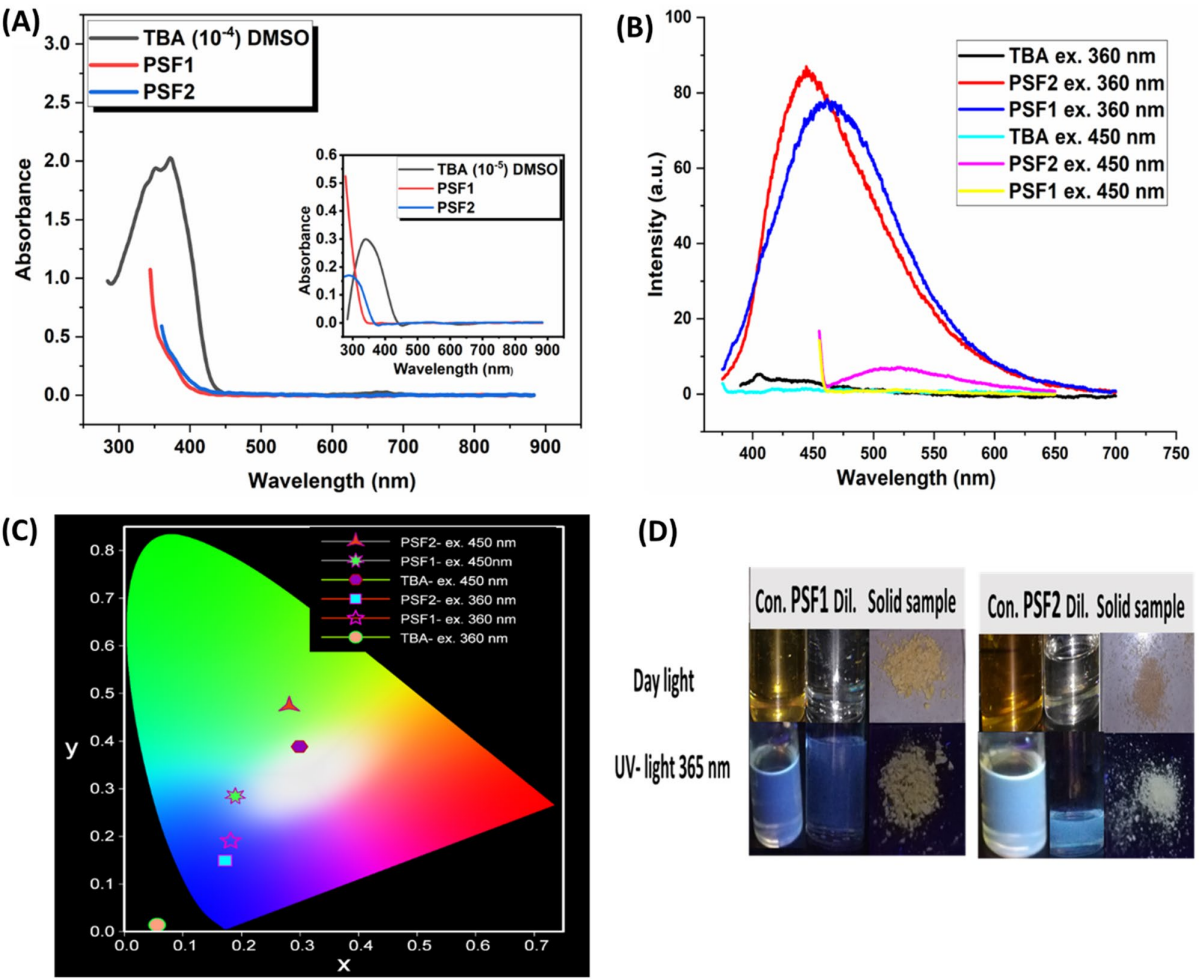
(**A**) UV–Visible absorption (10^−4^ of TBA, 1 mg in 10 ml DMSO of polymers), inset UV absorption of diluted solution (10^−5^ of TBA, 0.1 µg in 10 ml DMSO of polymers). (**B**) PL emission spectra of TBA and polymers at (λ_exc._ = 360 nm, λ_exc._ = 450 nm). (**C**) CIE diagram of (**B**). (**D**) Images of solid, diluted (1 mg), and concentrated (0.5 g) in DMSO solution of PSF1 and PSF2 under daylight and UV light excitation.

The photophysical properties of polymers are checked through UV–visible absorption and fluorescence emission. Figures 7a, S8 introduces the UV- absorption spectra and calculated bandgap from Tauc's equation. The samples are measured in DMSO solution of TBA, PSF1, and PSF2, with concentrations (10^−5^, 10^−4^ TBA and 0.1 µg, 1 mg in 10 ml for each polymer). The TBA molecule exhibits a maximal absorption band at 350 nm; this band is unique to that molecule. Its bandgap energy looks to be approximately 2.9 eV due to the amide group's (n-π^*^) transition and the benzene ring's (π-π^*^) transition. Polymers show a broad tail of absorption band extending into the visible region for both (π-π^*^) transition of aromatic and (n-π^*^) transition around (278 nm, E_g_ = 3.9 eV) for PSF1, (299 nm, E_g_ = 3.2 eV) for PSF2, the blue shift of the peaks and lower values of energy band gaps of the very diluted solution of polymers is due to that these samples do not absorb in the near UV. This remarkable absorption is due to the aggregation and hydrogen bonds between molecules; this appears clearly in PSF1, which has negligible absorption; this is attributed to the intramolecular interaction of hydrogen bonds inside the unit structure, leading to that decrease in the wavelength^61,62^.

The fluorescence behavior of TBA and the prepared polymers is displayed in (Fig. 7b, c and d). TBA molecule has various behaviors emission at two different excitation wavelengths. The first excitation wavelength at λ_exc._ 360 nm of 10^−4^ solution of TBA in DMSO is absorbed by the molecule and does not show any emitted color (appears as a black object); this appears clearly from chromaticity diagram CIE, its quantitative color coordinate (x, y) = (0.056, 0.014). In contrast, the same solution emits white light with CIE coordinates at a wavelength of around 450 nm (0.299, 0.388). This one is accomplished by combining blue and yellow colors (complementary hues), which may result from excited-state intramolecular proton transfer (ESIPT). This bond aroused between the hydrogen of the nitrogen atom and oxygen of the carbonyl group (amide group), forming tautomer form, leading to a decrease in the balance between conjugation and aromaticity of the benzene ring and this appears clearly from quantum calculations which show the small bandgap value for that molecule. So the equilibrium is established in the excited state between normal and tautomer forms generating white light emission^63,64^.

For polymer PSF1, diluted solution in DMSO (1 mg in 10 ml solvent) is excited with wavelength λ_exc._ 360 nm, blue emission at λ_em_ = 440 nm is observed with CIE coordinates (0.181, 0.190) and low emission intensity compared to the rest polymers when they are excited at the same wavelength. While excitation of the same solution at a longer wavelength λ_exc._ 450 nm exhibits no emission, and its quantized CIE coordinates (0.189, 0.285) display that the polymer has greenish-blue emission near to white light emission, which is appeared clear in Fig. 7d, the photo of its concentrated solution (5 mg in 10 ml DMSO) under UV emission 365 nm gives a white light emission. This emission may be due to the intermolecular hydrogen bonding that decreases the bandgap because of the molecule's restricted rotational and vibrational levels.

On the other hand, the polymer PSF2 possesses similar behavior in different solution concentrations when they are excited with different wavelengths. Excitation of (1 mg in 10 ml DMSO) solution with wavelength λ_exc._ 360 nm resulted in blue emission with higher intensity than PSF1 at λ_em._ 445 nm with CIE coordinates (0.172, 0.149). While its excitation at λ_exc._ 450 nm exhibits green emission at λ_em._ 510 nm with CIE coordinate PSF2 (0.281, 0.473). The concentrated solution (5 mg in 10 ml DMSO) has a white light color, and the solid sample of PSF2 shows a clear white light emission under UV irradiation λ_exc._ 365 nm, this distinct attitude of emission-dependent excitation is attributed to the crosslinking enhancing emission CEE that is higher in PSF2 than PSF1^65–67^. A (TBA) unit in PSF2 imparts this behavior in contrast to PSF1, which does not contain a TBA unit in its chain; this is clear from quantum calculations that the PSF2 unit has a lower bandgap which results from intermolecular hydrogen bonding and higher crosslinking. Introducing a multi stimuli response MSR in these molecules for several optoelectronic applications.

This unique fluorescence behavior of these polymers makes them a good chemical tool for colorimetric sensing of metal ions. Figures S9, 8, and Table S3 show the UV–visible absorbance, fluorescence emission, and CIE diagrams of (1 mg of polymers and 1 mg of metal ions in 10 ml DMSO as solvent). The UV absorbance data exhibit that PSF1 with metals (Zn^+2^, Cu^+2^, Ni^+2^, Co^+2^) results in a small redshift of the absorbance band, while PSF2 with the same metals displays a small blue shift of the absorbance band. For Fe^+3^ ion, all polymers solution has a large redshift in the absorbance band. This shift may be because polymer PSF2 has more conjugation and donation than PSF1 in its structure. When they bind with metals, their donation decreases and produces a blue shift maintaining the same spectrum feature. Fluorescence emission of PSF1 is greatly affected by different binding metals, the intensity of the emission is highly increased with Co^+2^ ion than the other metals and also shows a blue light emission with CIE coordinate (x,y) (0.162, 0.104), Zn^+2^, and Ni^+2^ have higher intensities than the polymer alone and blue light emission, Cu^+2^ almost the same emission intensity as PSF1 with a little difference and Fe^+3^ has a very small intensity in the blue region. According to the emission intensity, their ordering is as follows: Co^+2^ > Zn^+2^ > Ni^+2^ > Cu^+2^ > Fe^+3^ so that PSF1 can be used as a metal ion sensor for all these metals except for the Cu^+2^ ion. The emission of PSF2 shows different behavior towards these metals, all metals solutions with PSF2 emit in the blue regions from values of CIE coordination in Table S3 but with different intensities. Co^+2^ ion solution has a higher emission intensity, but Zn^+2^ has the same as polymer, Ni^+^2, and Cu^+2^ solutions have lower intensities than polymer, and Fe^+3^ ion has no emission. The arranging intensities of emission are as follows: Co^+2^ > Zn^+2^ > Ni^+2^ > Cu^+2^ > Fe^+3^, and PSF2 as a metal ion sensor for all these metals except for Zn^+2^ ion is possible with different behavior from PSF1. This difference in chemo-sensitivity behavior is due to the difference in binding metals inside the internal structure of these polymers; PSF1 displays greater and intensifying emission difference resulting from binding of metals to the electron-withdrawing group EW reinforcing D-π-A system also with ligand metal charge transfer LMCT phenomena as its structure has lower conjugation than the other polymers^68^. In contrast, PSF2 exhibits metal–ligand charge transfer MLCT due to binding metals to electron-donating group ED, which decreases the conjugation and converting D-π-A system to A-π-A system^69^. The quenching and small emission of PSF2 are due to ESIPT^70,71^, which is fast compared to the fluorescence process and intersystem crossing^72^.

**Figure 8:**
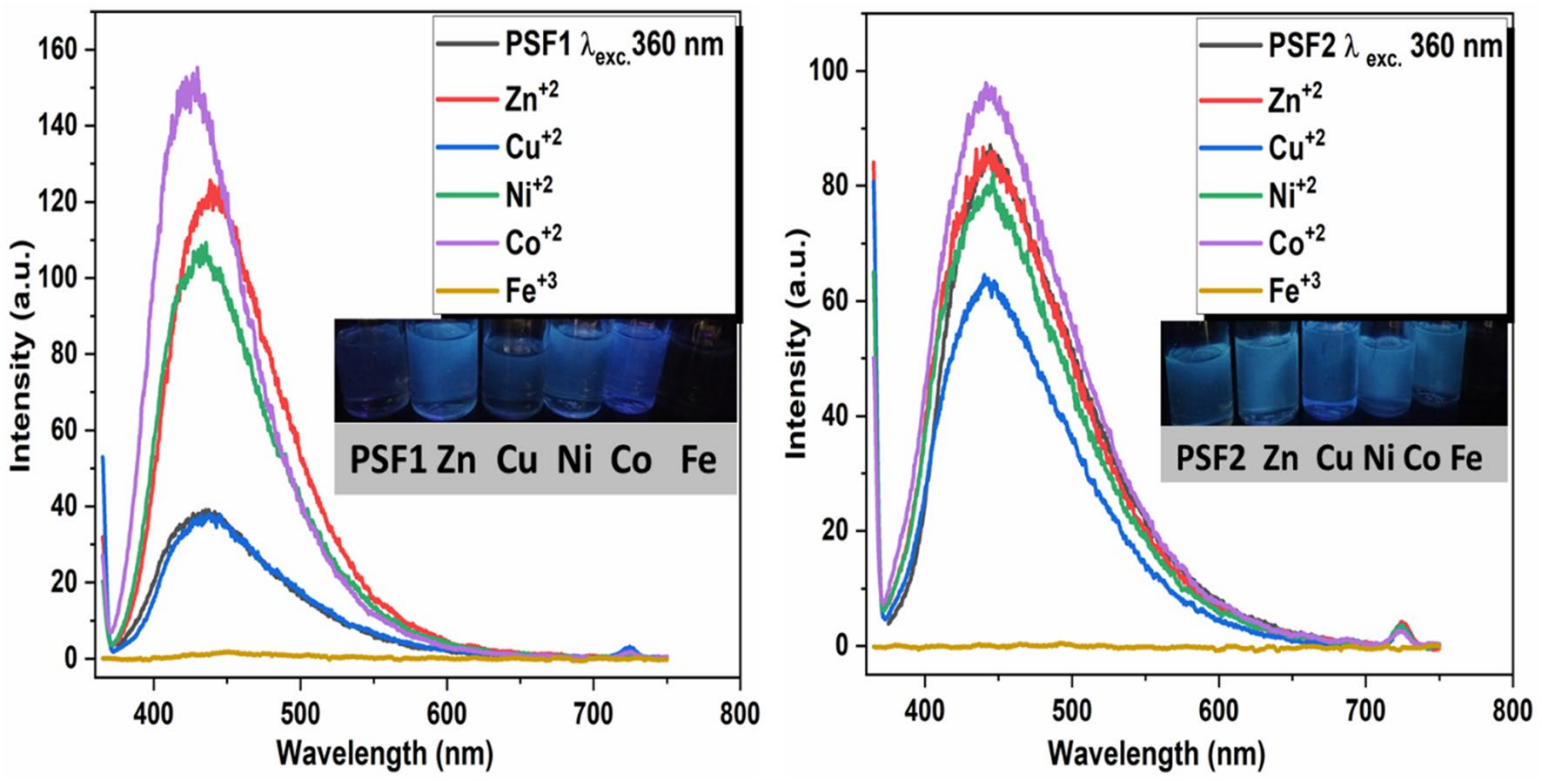
PL spectra of polymers and metals in DMSO with λ_exc._ 360 nm, inset images of polymers and their metals under UV lamp 365 nm.

The senstivity of the polymers toward these metals are tested through adding different concentrations ranging from (3–0.9 n gmol/ml) of (Zn^+2^, Ni^+2^, Cu^+2^, Fe^+3^, Co^+2^) to polymers solutions with concentration (3 n gmol/ml of PSF1 or PSF2). Figures S10–14 display the absorption intensities of these solutions and the relation between their different concentrations and intensity of polymer absorbance. Table S4 shows the standard deviation (σ) and slope (S) of linear relation between absorption intensities with metal`s concentrations to calculate the limit of detection of polymers (LOD) from equation (3.3σ/S) and limit of quntation (LOQ) from (10σ/S). LOD for PSF1 is (11.29, 6.42, 1.49, 0.7, 1.11 n gmol/ml) for (Zn^+2^, Ni^+2^, Co^+2^, Fe^+3^, Cu^+2^) respectively, which is sensitive for low concentrations of ferric metal. On the otherhand, LOD for PSF2 is (3.75, 2.72, 1.49, 1.33, 0.79 n gmol/ml) for (Zn^+2^, Ni^+2^, Co^+2^, Fe^+3^, Cu^+2^) respectively, which is sensitive for low concentrations of Cu^+2^ metal. From these results PSF2 is more sensitive to these metals than PSF1 due to its higher chelating ability than PSF1. Also, these polymers become more applicable in detection of these metals due to the lower detection values of these polymers.

### Electrochemical study

The previous results demonstrated the ability of these polymers to connect with diverse metals through chelation mechanisms, particularly iron, due to their heteroatom-rich architectures. Their corrosion inhibition is being investigated, and researchers attempt to mitigate or prevent this environmental issue.

### Open circuit potential measurements (OCP)

**Figure 9:**
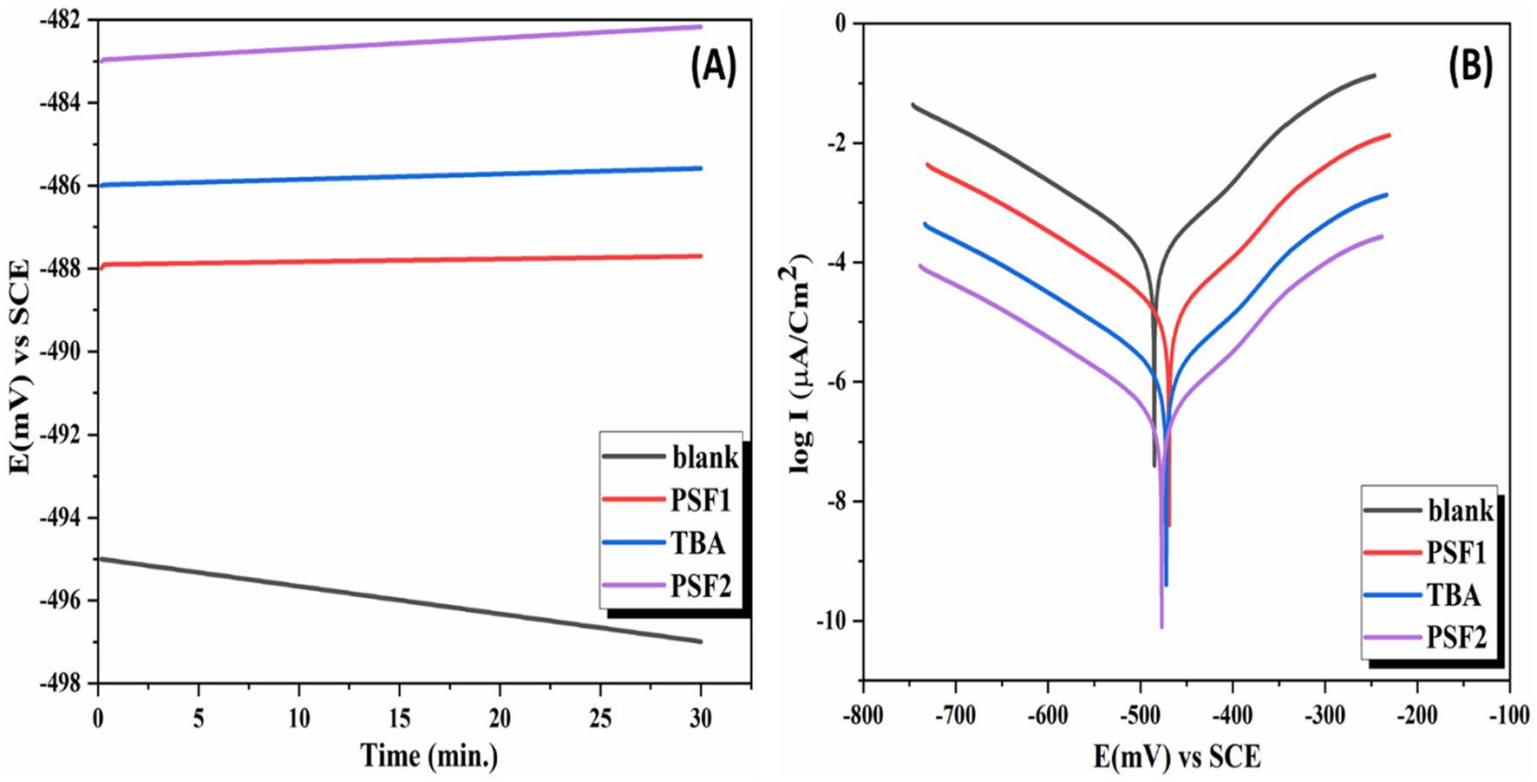
(**A**) Potential-time curves and (**B**) Tafel plots polarization curves of mild steel exposed to different inhibitors at 200 ppm for TBA, PSF1, and PSF2.

OCP means that no current passes in the electrochemical cell used for measuring the corrosion rate of metal dissolution, the measurements of the polarization curves are initiated after submerging the employed mild steel electrode inside the prepared solution to estimate the steady-state potential (E_S.S_). Figure 9a displays each E_im_ immersion and E_S.S_ of mild steel exposed to an acidic medium with and without inhibitors between + 20 and − 20 mV vs E_S.S_ for linear polarization (LP). It is clear from E_S.S_ of blank that there is a slight shift from a negative to a positive direction of potential (492–490 mV) for a blank solution, indicating the formation of oxide film on the mild steel surface. Pickling the same electrode in a solution containing the acidic medium with the tested samples also shifted the E_S.S_ value to a more positive potential than the blank solution, suggesting that the inhibitor molecules blocked the active anodic and cathodic sites on the metal surface. Table S5 shows E_S.S_ values of OCP for each inhibitor used in this study, and all values are moved to more positive potential than the blank solution.

### Polarization measurements

Tafel plot polarization measures the corrosion current, potential, rate, and inhibition efficiency of mild steel exposed to acidic media with and without inhibitors within the range − 250 to + 250 mV vs E_S.S_ and a scan rate between 0.166 and 0.4 mV/S. The inhibitor's efficiency percent (IE%) and the corrosion rate (CR) are calculated from Eqs. (1) and (2)^73^.

1 $${\text{C}}.{\text{R}}\left( {{\text{mpy}}} \right) = 0.13{\text{ I}}corr\frac{{\left( {Eq.Wt} \right)}}{A.d}$$

where corrosion rate ${\text{ C}}.{\text{R in }}\left( {\text{millimetres per year}} \right)\left( {{\text{mpy}}} \right)$, the quantity of corrosion current ${\text{Icorr}}$ in (μA/cm^2^), the metal equivalent weight in $\left( {{\text{Eq}}.{\text{Wt}}} \right)$ (g/eq), area A (cm^2^), density d in (g/cm^3^), and time conversion factor 0.13 metric.

2 $${\text{IE \% }} = \left[ {\frac{{\left( {{\text{C}}.{\text{R uninh}} - {\text{C}}.{\text{R inh}}} \right)}}{{{\text{C}}.{\text{R uninh}}}}} \right]$$

where corrosion rates without or with inhibitors $\left(\mathrm{R uninhn},\mathrm{ C}.\mathrm{R inhn}\right)$, respectively.

Figure 9b introduces Tafel plots (E mV against log I µA/Cm^2^) of mild steel with or without the inhibitors in an acidic medium; the figure demonstrates the anodic and cathodic curves of the inhibitors proceed in the positive direction more than the blank curve. Parameters of Tafel polarization are presented in Table 2. From these results, the corrosion rate C.R decreases as the inhibitors are applied to the mild steel and are arranged from the highest corrosion rate amount to the smallest value as follows: blank > PSF1 > TBA > PSF2 (the best inhibitor); this means that a higher IE% is for PSF2. These investigational results agree with theoretical quantum calculations; Table 3 exhibits parameters of these calculations by Koopmans' theory 80 electronegativity χ, hardness η and softness σ, which help manifest the interactions between metal–inhibitor. From this theory, − *E*_LUMO_ corresponds to the electron affinity (*A*), and − *E*_HOMO_ is equal to the ionization potential (*I*); these values A and I are used to deduce the chemical properties of the molecules. They all declare that PSF2 is the best inhibitor, as it has the smallest energy difference with higher softness, electronegativity, and lower hardness. Hence, it is more active (less stable) and has the highest electron-donating and electron-accepting ability. It is known that the inhibition mechanism of the mild steel surface is attributed to the formation of the protective layer of the inhibitor (good electron donor or electron acceptor)^74^, which retards the attack of corrosive media on the steel surface. This inhibition is the highest in polymer samples owing to their complexation with the metal surface, especially if they contain hetero atoms in their structure as O, S, and N atoms^34–36^. PSF2 has a high degree of crosslinking and D-π-A, while PSF1 does not have TBA in its structural unit responsible for this reactivity and thus lower inhibition efficiency^75^. Corrosion potential values E_corr_ for these inhibitors are in the noble direction; the difference between their values and E_corr_ of the blank does not exceed 85 mV, indicating that these inhibitors act as mixed-type inhibitors.

**Table 2:** List of potentiodynamic polarization descriptors for mild steel exposed to 1.0 M H_2_SO_4_ without and with different inhibitors.

Media	Rp (Ω)	Ecorr(mV)	Icorr (µA/Cm^2^)	C.R(mpy)	IE%
Blank solution	50	497	3250	2997	–
PSF2	520	481	260	240	92
TBA	215	486	700	645	78
PSF1	187	489	786	725	76

**Table 3:** Parameters of quantum chemistry of the molecules calculated from E_HOMO_ and E_LUMO_ values by Koopmans theory.

Molecule	I (eV)	A (eV)	*η (eV)*$\frac{ = I - A}{2}$	σ (eV)^−1^ (= 1/η)	χ (eV)$= \frac{I + A}{2}$
PSF2	0.2485	0.3033	0.0274	36.5	0.2759
TBA	0.2542	0.3438	0.0448	22.3	0.299
PSF1	0.1950	0.3186	0.0618	16.18	0.2568

### Measurement of dye adsorption

The adsorption of two different types of charged dye molecules, cationic and anionic, in a basic medium can be used to investigate the nature of these polymers' surfaces and the porous characteristic that plays a part in the corrosion inhibition outcomes^76^. These dye molecules are a major environmental issue in aquaculture systems. The change in watercolor indicates its contamination and pollution, the most popular pollutants that cause a color variation are organic dyes. These dyes are stable, toxic, and highly soluble in water; they result from industries like dyeing, textile, paper, and pulp^77^. Their discharging in water bodies harm the aquatic system through their tendency to absorb light, preventing it from penetration and hindering the photosynthesis process, affecting the biota and living organisms in the water. There are numerous techniques to eliminate these contaminants from the waterbody, for instance, adsorption, coagulation, degradation, and inverse osmosis^78,79^; between them, the simplest, easiest, and more effective process is the adsorption procedure. Cationic dye Methylene blue (MB) is utilized in an extensive range of applications as indicators and biological dyeing, detected in wastewater, and is recognized for its stability towards biodegradation^80^. An anionic azo dye found in the food and drug industries is Sunset yellow (SY), so its existence in the aquatic system is found continuously.

The prepared polymers are tested for their adsorption of these dyes' solutions, which have a concentration (2 × 10^−5^) in a basic medium at (pH = 9 for MB and pH = 13 for SY). The capacity of dye adsorbed (q mg/g) is calculated quantitatively from Eq. (3):

3 $${\text{q}} = \frac{{\left( {{\text{C}}0 - {\text{Ct}}} \right){\text{*V}}}}{{\text{m}}}$$

where the adsorbed solution volume V in (L), the weight quantity of adsorbents polymer m in (g), the original concentration C_0,_ and the remaining concentration C_t_ in (mg/L) of the dye solution. Calculation of dye removal percent is from Eq. (4):

4 $${\text{\% dye removal}} = \frac{{\left( {{\text{C}}0 - {\text{Ct}}} \right){*}100}}{{{\text{C}}0}}$$

**Figure 10:**
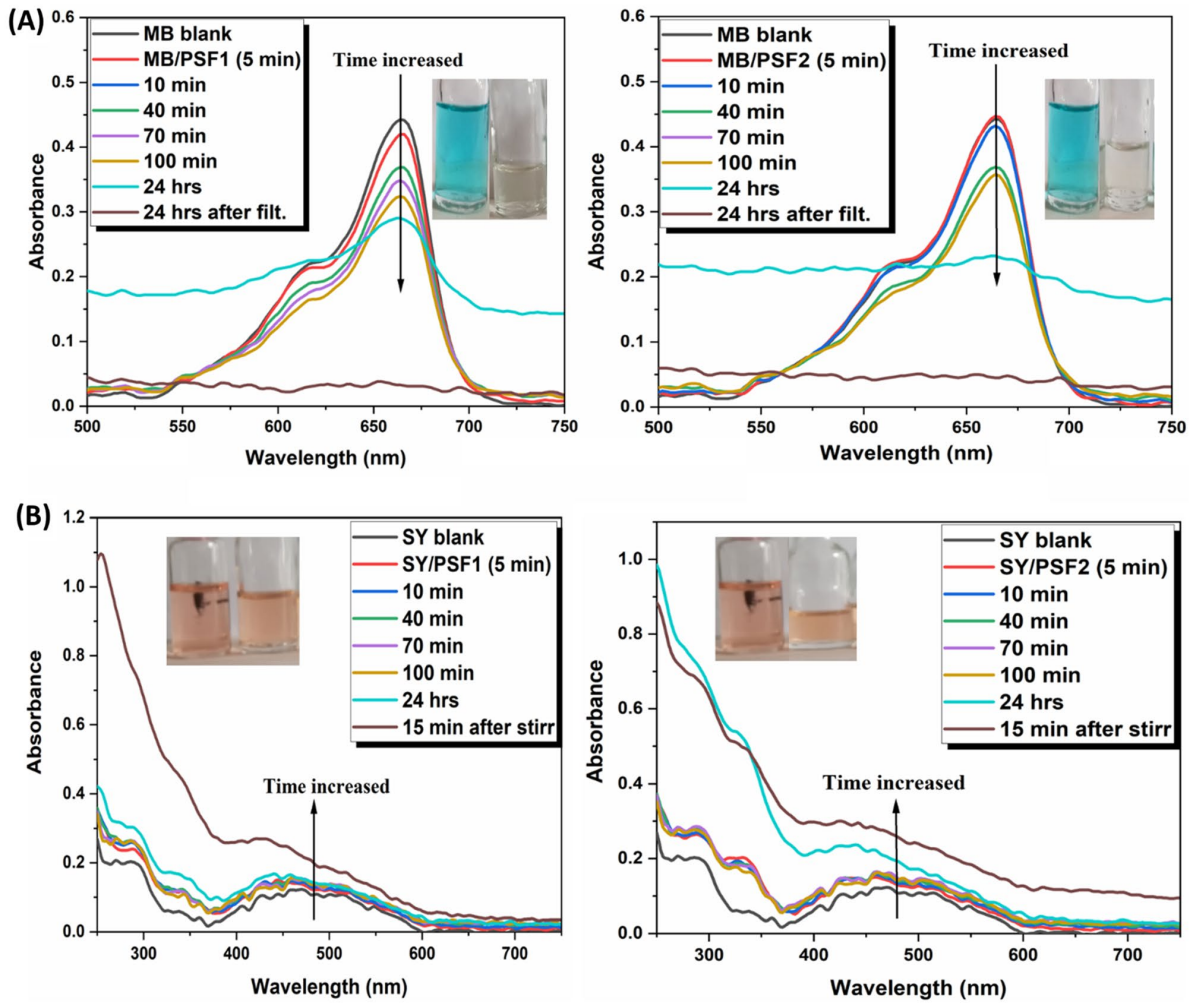
UV–Vis spectra of (**A**) single (MB) solution and (**B**) (SY) solution adsorption recorded at various times for PSF1 and PSF2, inset images are of solution left before and right after adsorption for each one.

Figures 10, S15, and Table S6 represent the data of the recorded adsorption behavior of these polymers, about (5 mg of them in 10 ml dye solution); calibrations curves of MB and SY are in Fig. S15a,b to determine the resulted concentration of solution at various time. MB solution has an absorption peak at 664 nm at pH 9 due to its single-molecule (MB^+^); decreasing the intensity of this peak indicates decreasing concentration. Figure 9 displays the graphs of adsorption of MB solution by polymers at various time intervals, and Fig. S9c represents their adsorption behavior after stirring for 15 min and after filtration. Two results are obtained depending on the way of removal; at various times without stirring, there is a gradual decrease in the concentration of MB solution for each one.

The PSF1 graph displays that the peak of MB decreases gradually. After 24 h, the peak of MB becomes broader and disappears after solution filtration with dye removal of about 96%, the same result of broadening the peak of MB but with a higher concentration and lower dye removal of about 59% is obtained when PSF1 is stirred in dye solution for 15 min. PSF2 performs the same manner as PSF1 but with higher proficiency in both ways after a long time and a short time with stirring, reaching a higher removal % of about (99.5%) after 24 h and 93.5% after stirring for 15 min. Dye molecules are adsorbed from the solution via electrostatic and diffusion processes, which rely on the conformation and size of these molecules. Small adsorbent pores are blocked rapidly by large molecules and accumulated at their surface, preventing more diffusion and adsorption^81^. Prepared polymers have a negative surface that attracts a cationic charge of MB dye. Their adsorption is processed according to previous mechanisms as small pores blocked rapidly, which is already existed in mesoporous PSF2 with a higher surface area from Brunauer Emmett Teller (BET) (Fig. S16), about 32.24 m^2^/g and an average pore radius of 2.4 nm and PSF1 with a smaller surface area about 28.79 m^2^/g and a pore radius 3.72 nm. PSF2 tendency of adsorption from Table S6 is smaller than PSF1 at various intervals. Still, after a long time or after stirring, its efficiency increases and reaches the highest one of them. Its reactivity from quantum results and higher surface area are the reasons for this feature, as in its graphs of adsorption after a long time or stirring, the peak of MB is decreased and becomes broad similar to the peak resulting from the polymerization of MB electrochemically verifying the polymerization of MB molecules on its surface, and the same process occurs in PSF1^82,83^. This polymerization process is higher in PSF2 than in PSF1 due to the higher surface area that allows more molecules to become more closely, decrease their electrostatic attraction via complexing with polymer, prefer polymerization through N(CH_3_)_2_ groups and so facilitate their removal from the solution after filtration^84^.

In the case of anionic dye SY, the adsorption mechanism is different from the cationic dye MB solution; the SY dye solution has a distinct absorption peak of about 443 nm in alkaline medium pH = 13. Figure 8b shows that at various time intervals of contact between polymer and dye solution and after stirring for 15 min, the intensity of absorption peak of SY dye is increased gradually with a small hypsochromic shift in both PSF1 and PSF2. The increasing intensity of PSF1 and PSF2 are high after a long time and after stirring. This increase in intensity has resulted from photoinduced electron transfer between dye molecules that are adsorbed at the surface of polymers, where they become close to each other, transferring the excited state from one to another. Their adsorption and charge transfer occurs through the weak hydrogen bond between anionic dye and hydrogen of secondary amine that existed in the polymer structure, so the higher crosslinking and assembling that exist in PSF2 make its charge transfer effect is smaller than in PSF1, which has lower assembling feature and allowing more hydrogen bond to be available to dye molecule^85,86^. Table 4 shows the adsorption capacities of different adsorbents^87–91^ that contain polysulfone, which indicates that the adsorption efficiency of these polymers is remarkable and effective in removing MB from solutions.

**Table 4:** The maximum adsorption capacities of PSF and other adsorbents for MB.

Adsorbents	Adsorption capacity (mg/g)	References
Polysulfone/hydrous ferric oxide NPs	13.2	^87^
Polysulfone/graphene oxide PSf/GO—1.25%	84.2% removal	^88^
Polyacrylonitrile Co Sodium Methallylsulfonate Copolymer (AN69) and Polysulfone (PSf)	75.75	^89^
Titanium oxide/polysulfone (TiNPs/PSF) composite	1000	^90^
PSf/S-UiO-66	48.06	^91^
PSF1	12.3	This work
PSF2	12.7	This work

The efficiency of the polymers and reusability are tested after four cycles, and shown in Fig. S17, Table S7. The desorption process is performed by washing the polymers with ethanolic solution, the adsorption capacity of polymers is decreased after the third cycle so these results suggest that PSF1 and PSF2 have a good adsorption capacity for MB dye after desorption.

## Conclusion

Poly (azomethine-sulfone)s are successfully prepared and investigated from barbituric acid and condensed structural monomer of the barbituric acid unit via condensation reaction and characterized by different techniques FT-IR, Raman spectroscopy, TGA, DSC, X-ray, and SEM. Their optical properties like UV–visible and fluorescence are checked, and they offer an interesting absorbance behavior leading to a different emission attitude. White light emission at a higher concentration has resulted, and blue light emission in the diluted solution, as a result of crosslinking enhancing emission CEE that is higher in PSF2 than PSF1 and the presence of TBA unit in PSF2. This emission feature is used as a metal ion sensor for different metals, showing a great sensitivity, owing to LMCT, MLCT, and ESIPT phenomena. Their pore size measurements state that they are mesoporous polymers, showing various routes toward different dyes molecules. Using these polymers as inhibitors are examined, they have a diverse and great inhibition efficiency depending on their structural formation. Quantum calculations for these compounds are examined, suggesting their structures in space, and matching all the experimental results.

## Supplementary Information

Supplementary Information.

## Data Availability

All data generated or analyzed during this study are included in this published article [and its supporting information files].
