# Supplementary material for: Synthesis, physico-chemical characterization, and environmental applications of meso porous crosslinked poly (azomethine-sulfone)s
Source: Sci Rep. 2022 Jul 27;12:12878. doi: 10.1038/s41598-022-17042-0 (PMC9329479; doi:10.1038/s41598-022-17042-0)
Supplement: Supplementary file 1 — Supplementary Information. [file 41598_2022_17042_MOESM1_ESM.pdf]

Supporting Information

Synthesis, Physico-Chemical Characterization, and Environmental Applications of Meso Porous Crosslinked Poly (azomethine-Sulfone)s.

Marwa M. Sayed [1,*],Mohamed Abdel-Hakim[2], Mahmoud H. Mahross [2] and Kamal I. Aly [3,*]

[1] Chemistry Department, Faculty of Science, the New Valley University, El-Kharja 72511, Egypt.

[2] Chemistry Department, Faculty of Science, Al-Azhar University, Assiut 71524, Egypt.

[3] Polymer Laboratory 122, Chemistry Department, Faculty of Science, Assiut University, Assiut, 71516, Egypt.

*To whom correspondence should be addressed

E-mail: Kamal Aly (Kamalaly@aun.edu.eg), Marwa Sayed (marwa.m@sci.nvu.edu.eg).

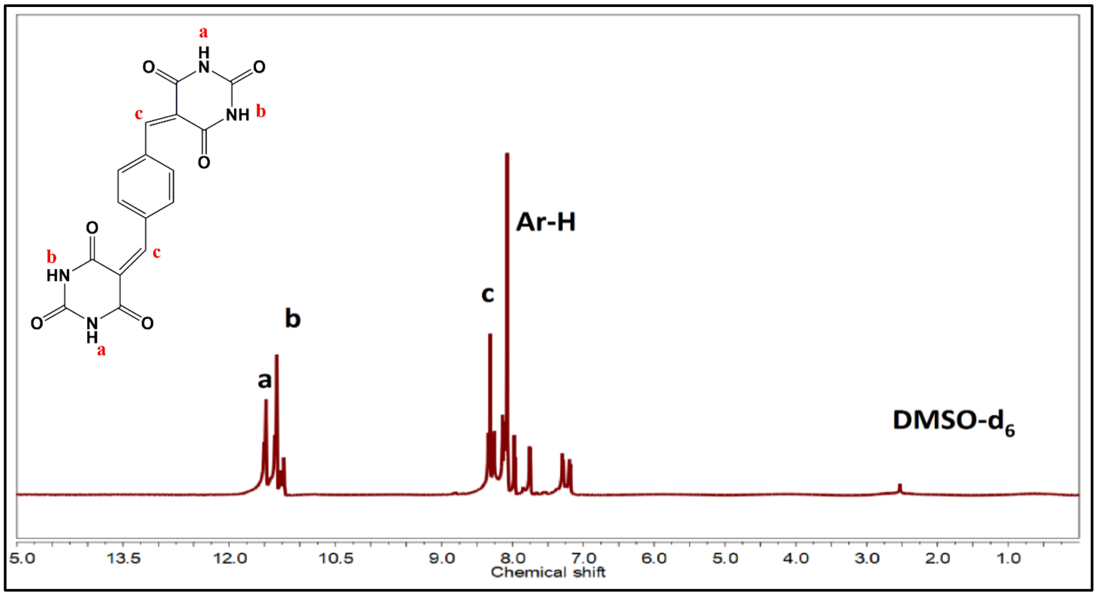

Figure S1. ^{1}H-NMR spectrum of TBA.

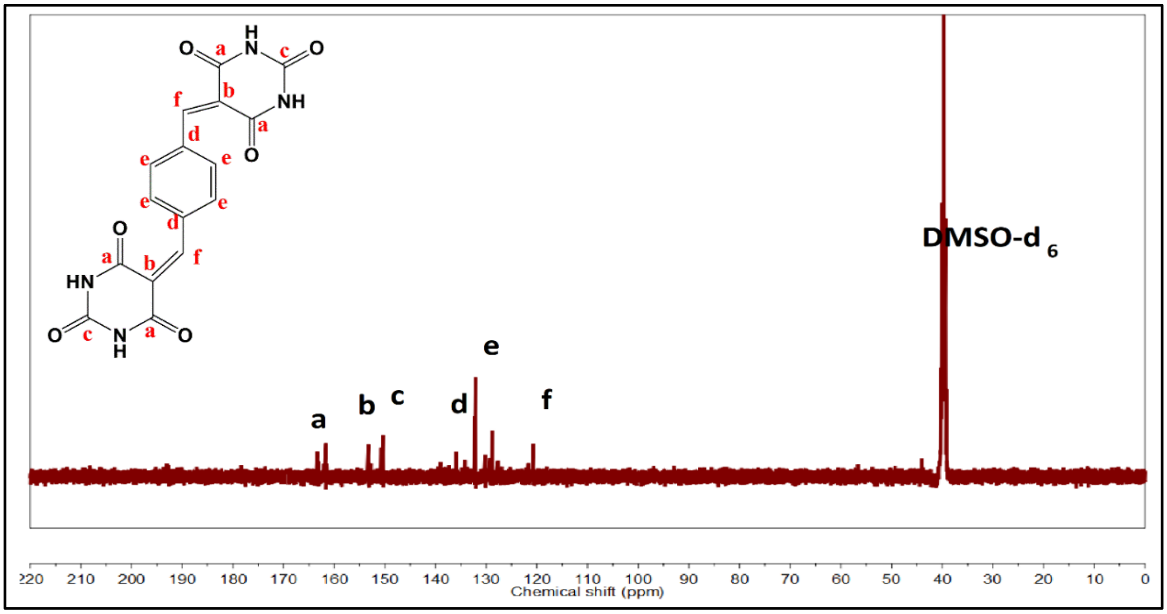

Figure S2. ^{13}C-NMR of TBA.

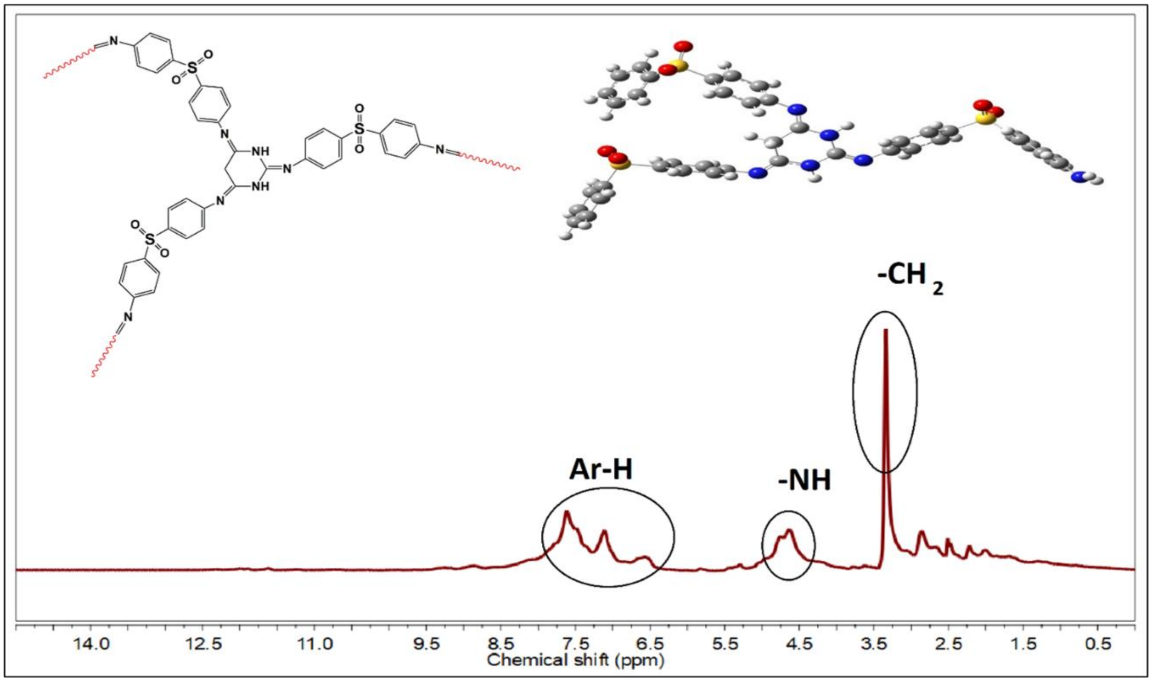

Figure S3. ^{1}H-NMR of PSF1.

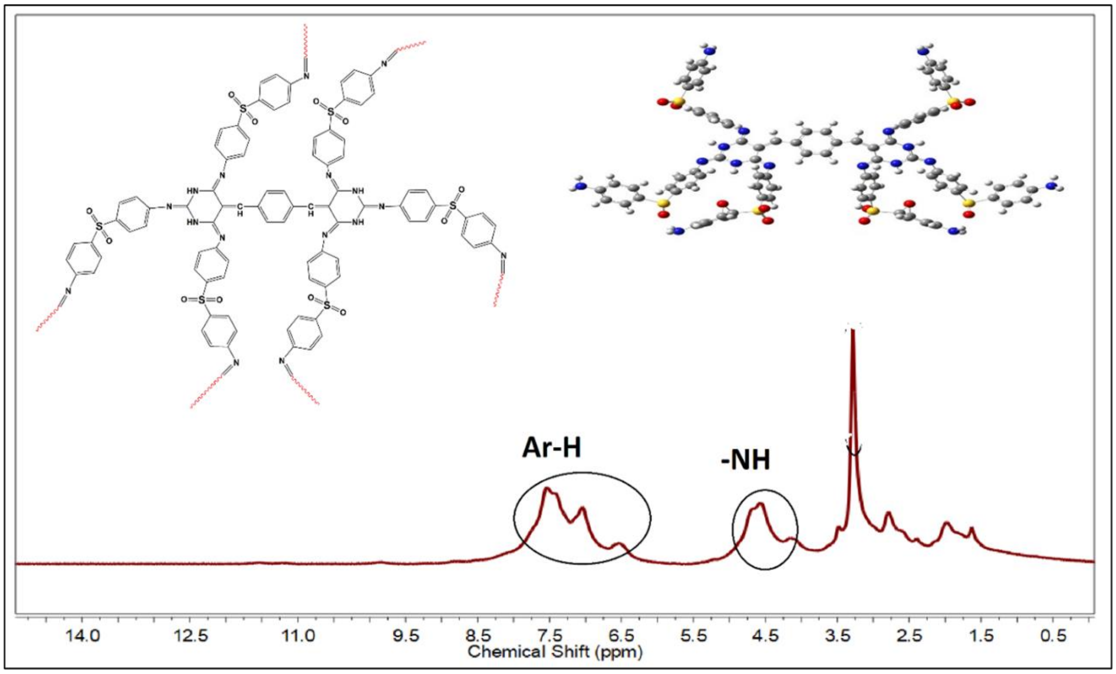

Figure S4. ^{1}H-NMR of PSF2.

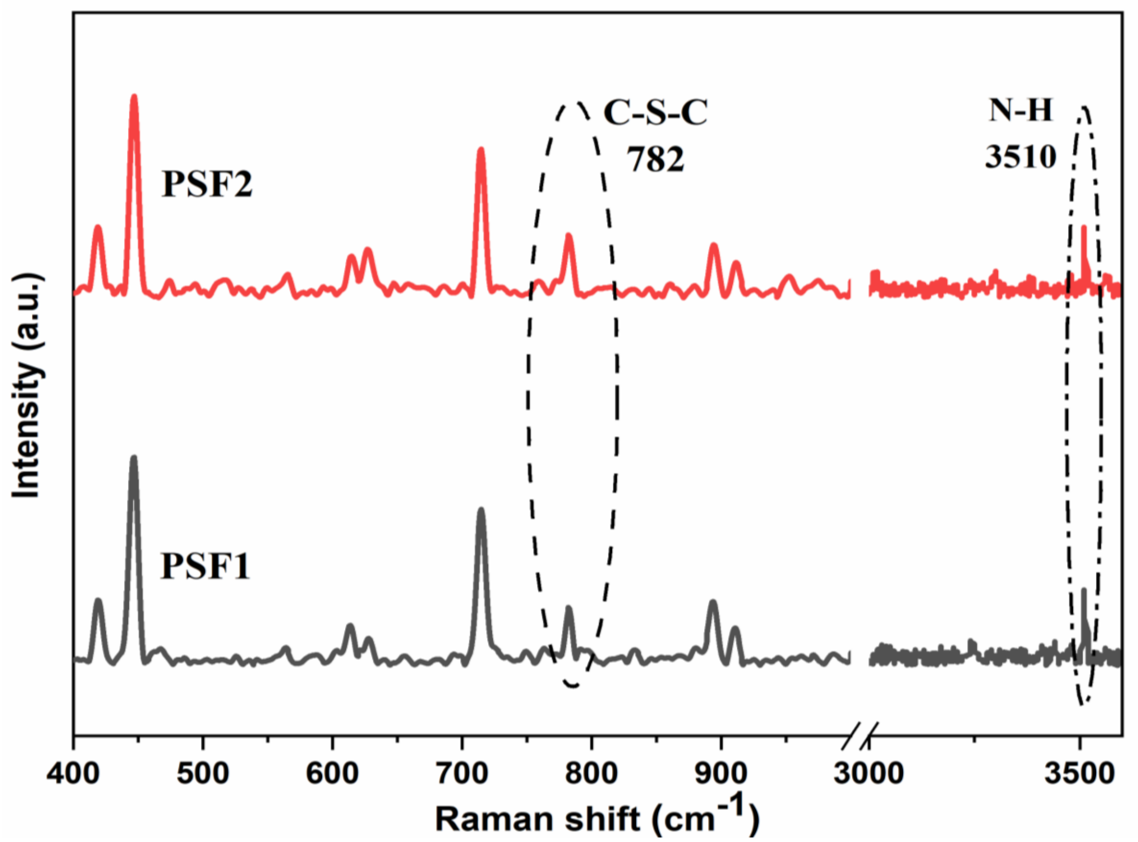

Figure S5. Raman spectra of PSF1 and PSF2.

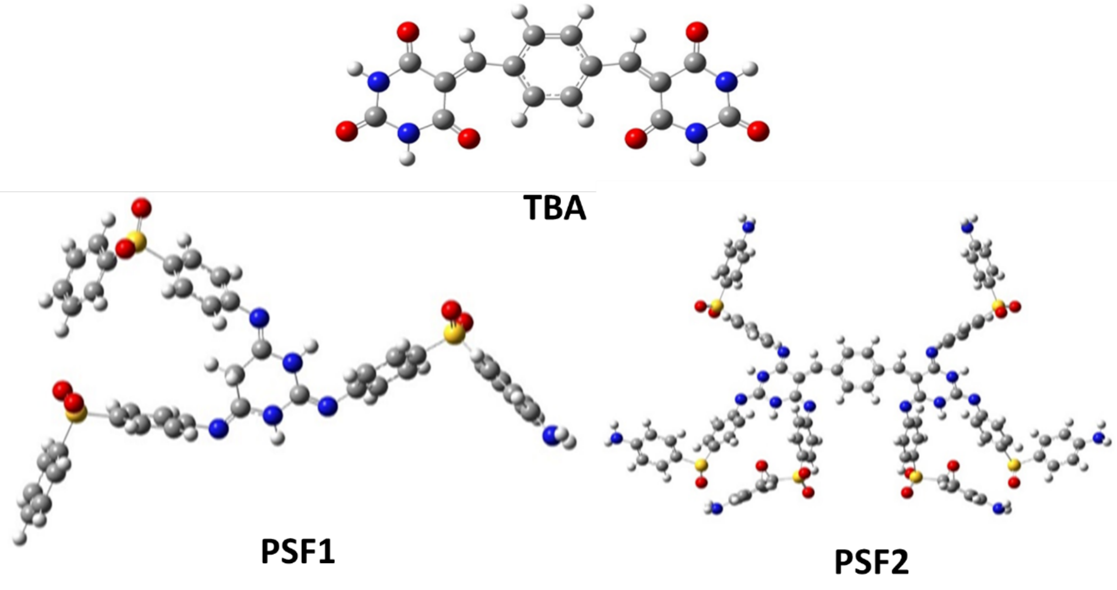

Figure S6. Optimized forms of TBA, PSF1 and PSF2 by using DFT, B3LYP/6-31G (d,p).

Table S1. E_{LUMO}, E_{HOMO} and energy gap for molecular system.

Structure	$-E_{LUMO}$ (Hartree)	$-E_{HOMO}$ (Hartree)	ΔE (E_{LUMO}-E_{HOMO}) (Hartree)
**PSF2**	**0.2485**	**0.3033**	**0.0548**
**TBA**	**0.2542**	**0.3438**	**0.0896**
**PSF1**	**0.1950**	**0.3186**	**0.1236**

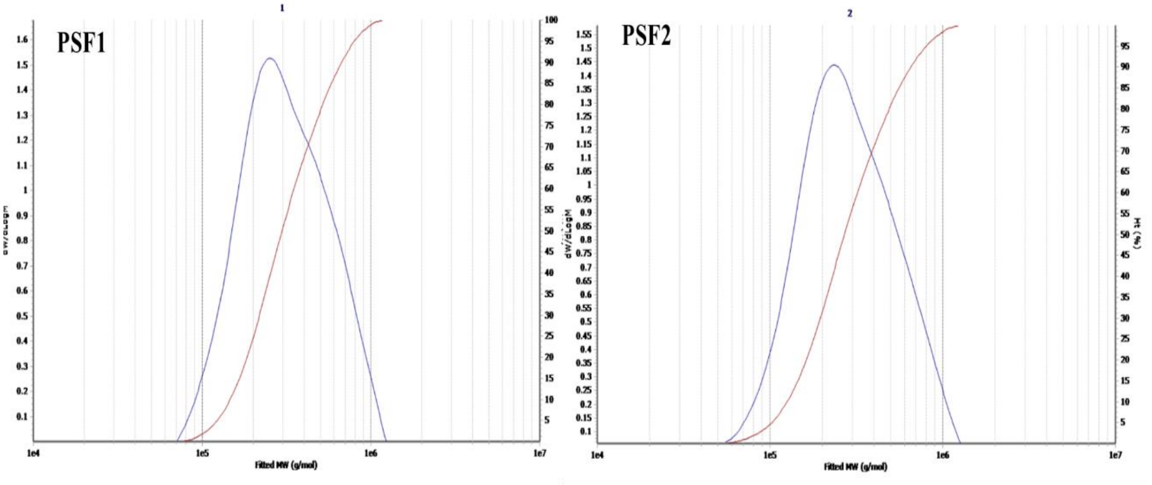

Figure S7. GPC curves of PSF1 and PSF2.

Table S2. M_W, number of repeating units and PD of PSF1, PSF2.

Polymer	M_W	M. wt of monomeric unit	N. of repeating units	PD
**PSF1**	**358619**	**396.51**	**904**	**1.37**
**PSF2**	**336628**	**658.31**	**511**	**1.46**

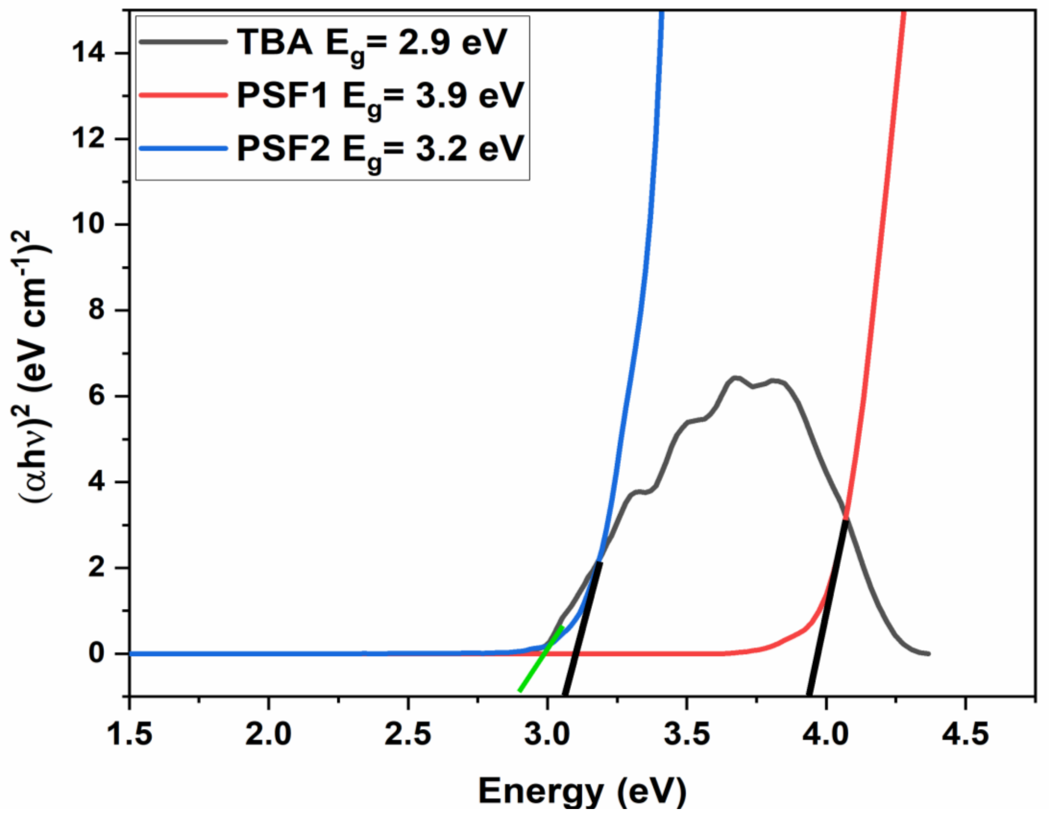

Figure S8. Tauc`s plots of TBA and polymers.

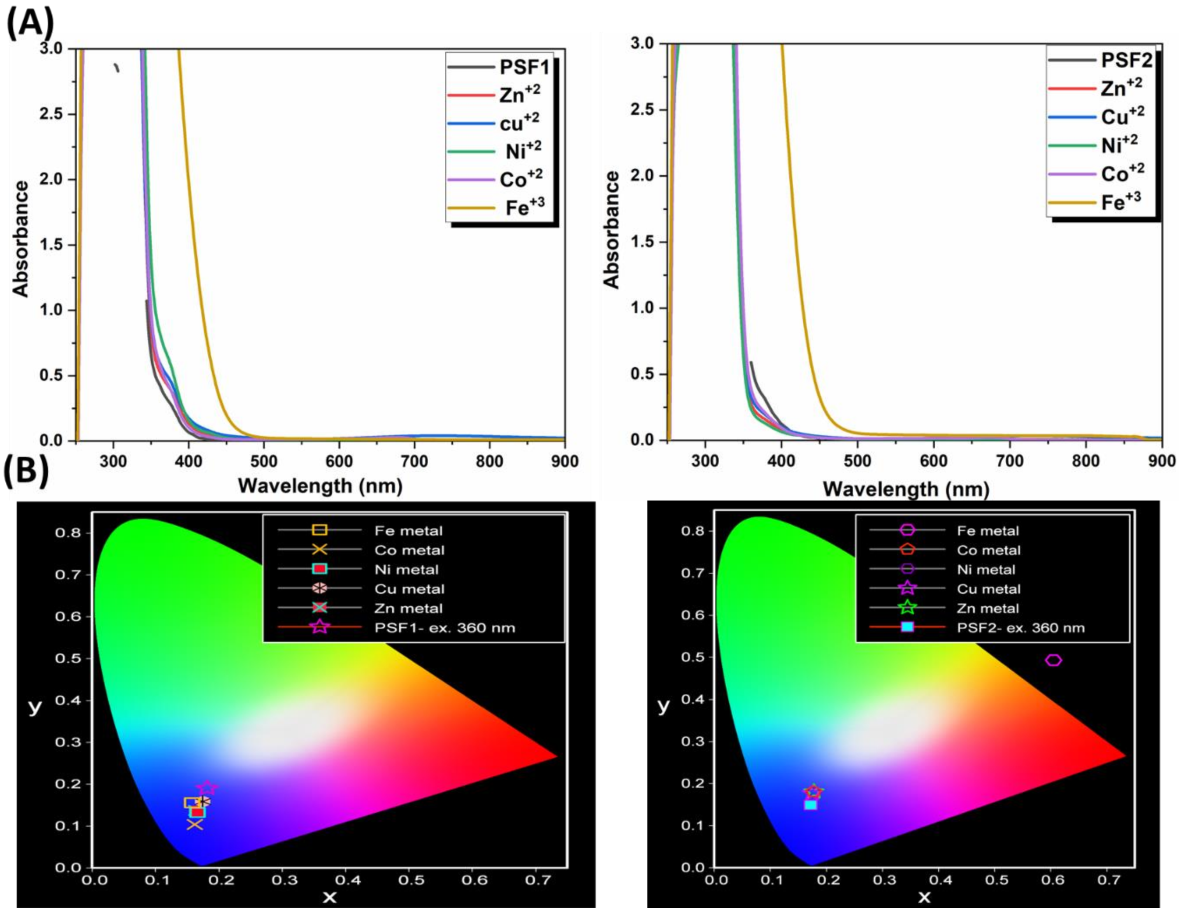

Figure S9. (A) UV-Vis absorption of polymers PSF1 and PSF2 with metals in DMSO with concentration (1mg in 10 ml solvent), (B) CIE chromaticity diagram of polymers PSF1, PSF2, and their metals ions (Zn^{+2}, Cu^{+2}, Ni^{+2}, Co^{+2} and Fe^{+3}).

Table S3. Values of emission CIE coordinates of TBA, polymers and their solution as metal sensors.

**Sample**	λ_{exc} **360 nm** (x, y)	λ_{exc} **450 nm** (x, y)
**TBA**	**0.056, 0.014**	**0.299, 0.388**
**PSF1**	**0.181, 0.190**	**0.189, 0.285**
**PSF1- Zn**	**0.164, 0.137**	
**PSF1- Cu**	**0.175, 0.159**	
**PSF1- Ni**	**0.166, 0.133**	
**PSF1- Co**	**0.162, 0.104**	
**PSF1- Fe**	**0.158, 0.156**	
**PSF2**	**0.176, 0.164**	**0.282, 0.474**
**PSF2- Zn**	**0.182, 0.215**	
**PSF2- Cu**	**0.178, 0.218**	
**PSF2- Ni**	**0.180, 0.213**	
**PSF2- Co**	**0.178, 0.207**	
**PSF2- Fe**	**-0.049, 0.228**	

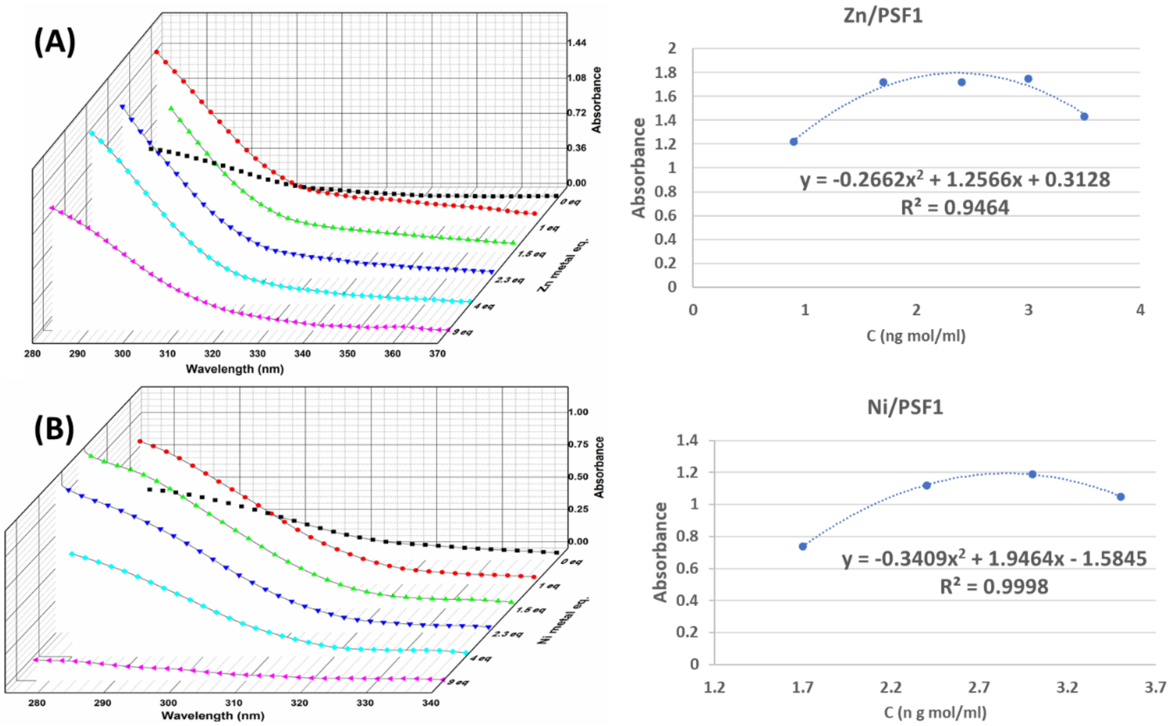

Figure S10. Various concentration of (a) Zn^{+2} in the presence of PSF1 (3 n gmol/ml) in DMSO, right figure UV/Vis spectra, left figure the absorbance intensity of PSF1. Various concentration of (b) Ni^{+2} in the presence of PSF1 (3 n gmol/ml) in DMSO, right figure UV/Vis spectra, left figure the absorbance intensity of PSF1.

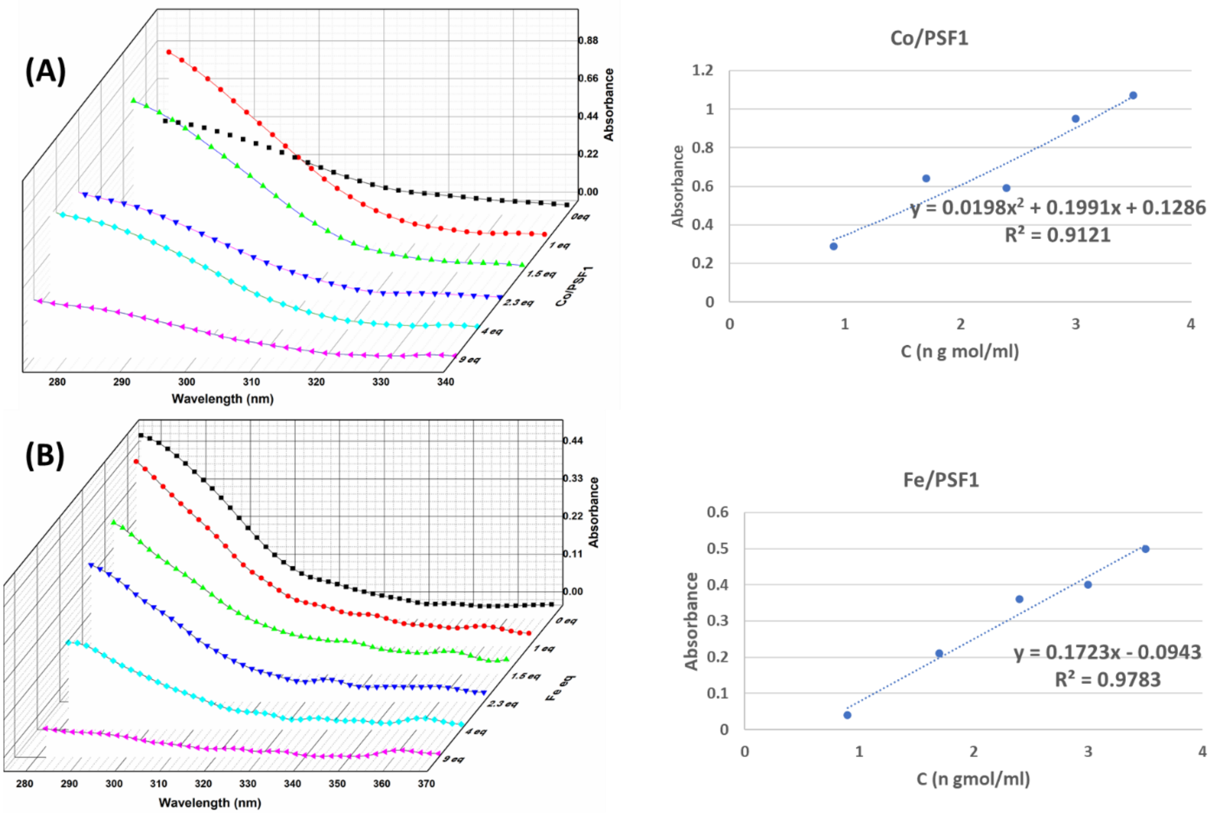

Figure S11. Various concentration of (a) Co^{+2} in the presence of PSF1 (3 n gmol/ml) in DMSO, right figure UV/Vis spectra, left figure the absorbance intensity of PSF1. Various concentration of (b) Fe^{+3} in the presence of PSF1 (3 n gmol/ml) in DMSO, right figure UV/Vis spectra, left figure the absorbance intensity of PSF1.

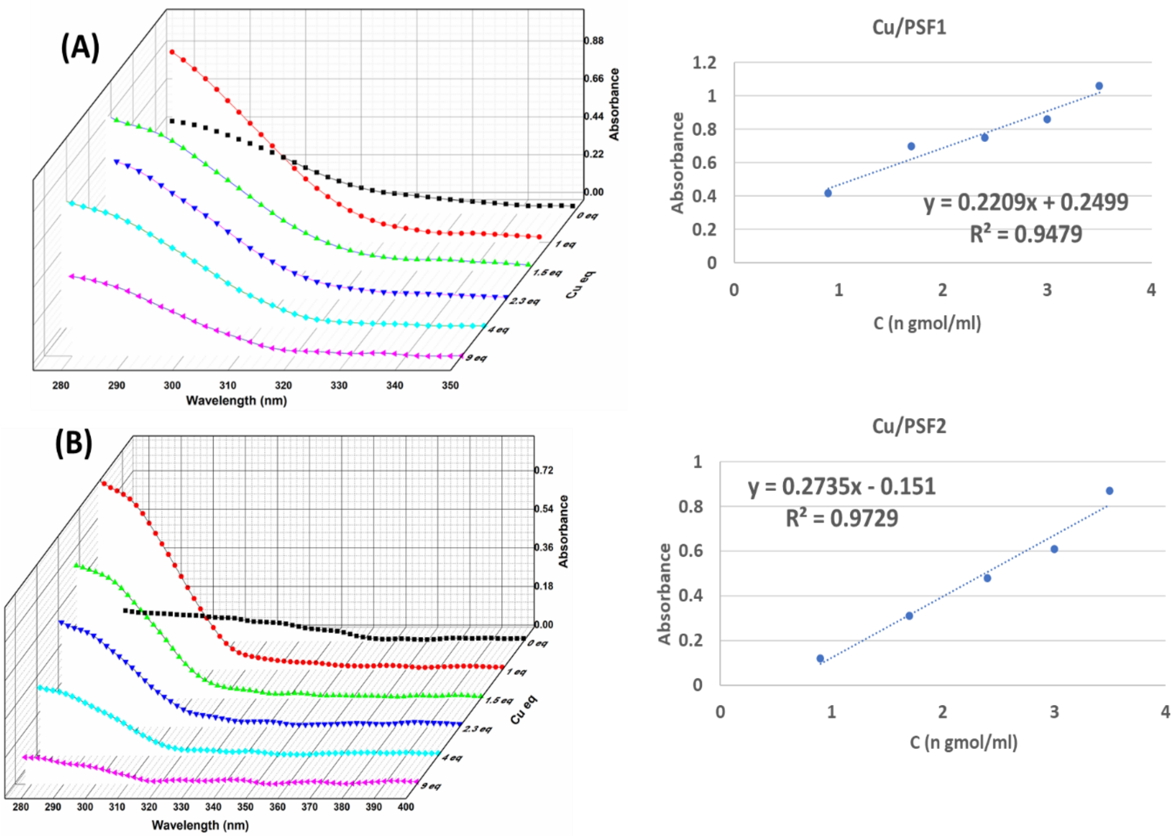

Figure S12. Various concentration of (a) Cu^{+2} in the presence of PSF1 (3 n gmol/ml) in DMSO, right figure UV/Vis spectra, left figure the absorbance intensity of PSF1. Various concentration of (b) Cu^{+2} in the presence of PSF2 (3 n gmol/ml) in DMSO, right figure UV/Vis spectra, left figure the absorbance intensity of PSF2.

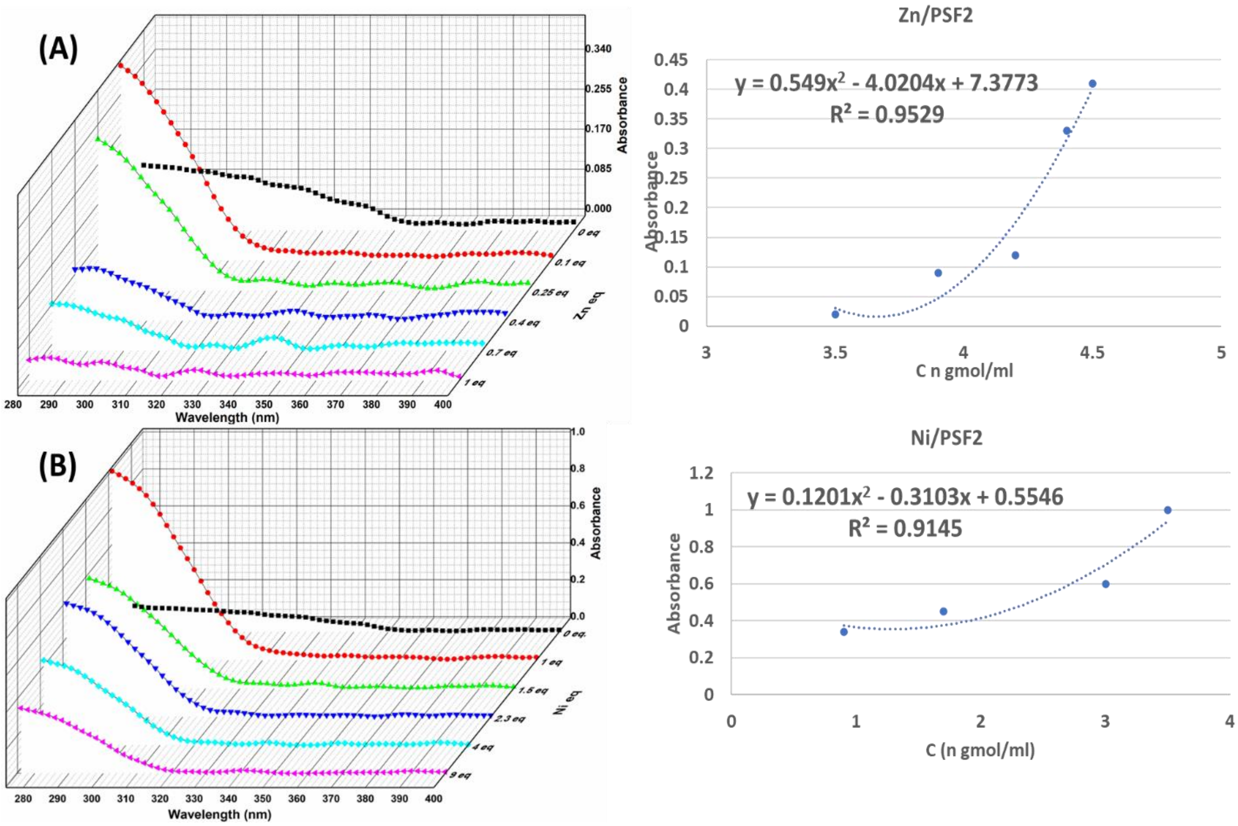

Figure S13. Various concentration of (a) Zn^{+2} in the presence of PSF2 (3 n gmol/ml) in DMSO, right figure UV/Vis spectra, left figure the absorbance intensity of PSF2. Various concentration of (b) Ni^{+2} in the presence of PSF2 (3 n gmol/ml) in DMSO, right figure UV/Vis spectra, left figure the absorbance intensity of PSF2.

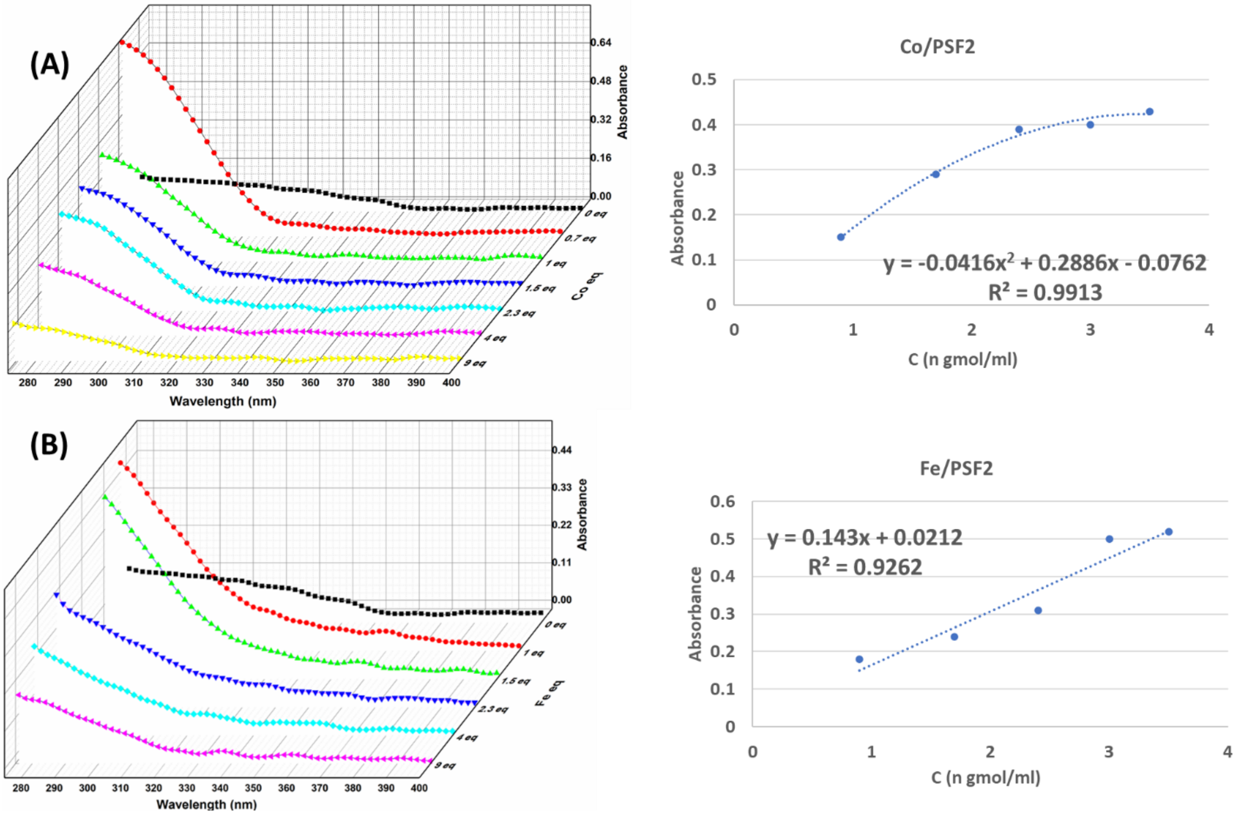

Figure S14. Various concentration of (a) Co^{+2} in the presence of PSF2 (3 n gmol/ml) in DMSO, right figure UV/Vis spectra, left figure the absorbance intensity of PSF2. Various concentration of (b) Fe^{+3} in the presence of PSF2 (3 n gmol/ml) in DMSO, right figure UV/Vis spectra, left figure the absorbance intensity of PSF2.

Table S4. standard deviation, slope, limit of detection (LOD), limit of quantitation (LOQ) of different metals (Zn^{+2}, Ni^{+2}, $Cu^{+2,}$ Fe^{+3}, Co^{+2}) with PSF1 and PSF2.

Metal	Standard deviation (σ)	Slope (S)	LOD	LOQ
Zn/PSF1	0.29964	0.08756	11.29	34.22
Ni/PSF1	0.35018	0.18011	6.42	19.44
Co/PSF1	0.129017	0.285915	1.49	4.51
Fe/PSF1	0.03676	0.17230	0.7	2.1
Cu/PSF1	0.07409	0.22089	1.11	3.35
Cu/PSF2	0.065337	0.27347	0.79	2.39
Zn/PSF2	0.42160	0.37121	3.75	11.36
Ni/PSF2	0.181377	0.21984	2.72	8.35
Co/PSF2	0.0477	0.10587	1.49	4.51
Fe/PSF2	0.057747	0.142958	1.33	4.04

Table S5. List of immersion and steady-state potentials for mild steel exposed to 1.0 M H2SO4 without and with inhibitors.

Media	$-E_{im}$ (mV)	$-E_{s.s}$ (mV)
Blank solution	495	497
PSF2	483	482.1
TBA	486	485.5
PSF1	488	487.7

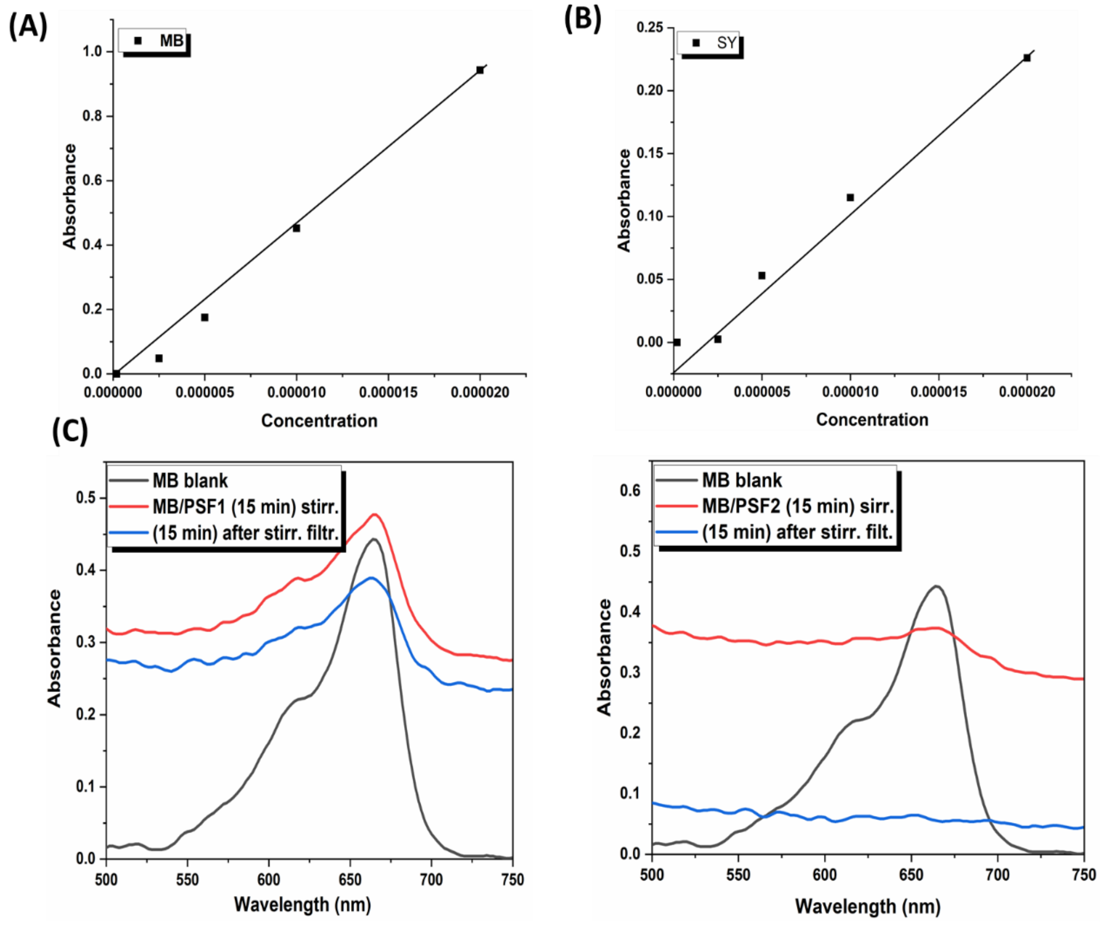

Figure S15. Calibration curve of (A) MB, (B) SY, and (C) UV–Vis spectra of (MB) solution adsorption recorded for PSF1 and PSF2 after stirring for 15 min.

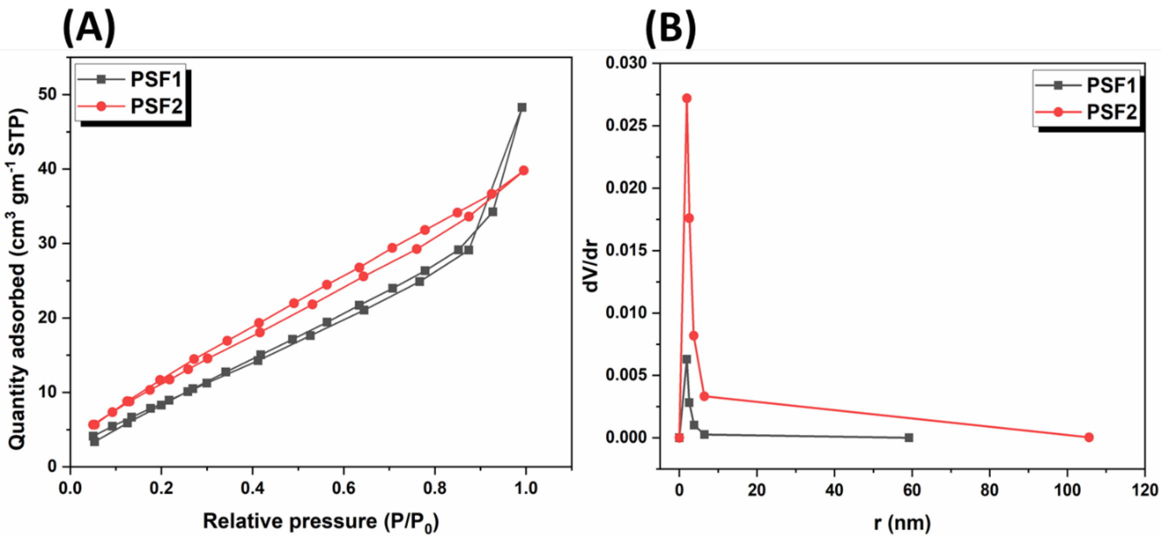

Figure S16. (A) BET isotherm, and (B) pore size distribution of polymers PSF1 and PSF2.

Table S6. Adsorption capacity (q) and dye removal % of PSF1 and PSF2 toward single (MB) dye solution.

Adsorbent	PSF1 q(mg/g)	% dye removal PSF1	PSF2 q(mg/g)	% dye removal PSF2
5 min	6.96	54.5	6.71	52.5
10 min	7.76	60.8	6.96	54.5
40 min	7.76	60.8	7.76	60.8
70 min	8.05	63	7.88	61.7
100 min	8.38	65.6	7.88	61.7
24 hrs	8.88	69.5	9.60	75.2
24 hrs after filt	12.3	96.3	12.7	99.5
15 min stirr	6.32	49.5	7.68	60.1
15 min after stir. filt.	7.62	59.7	11.9	93.5

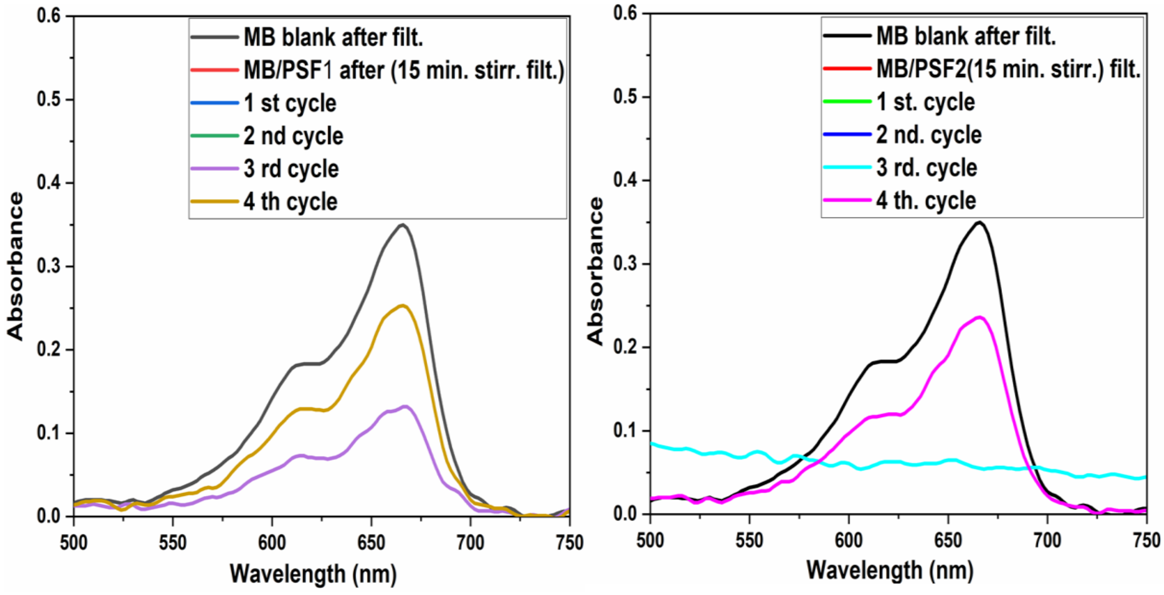

Figure S17. MB adsorption after 4 cycles for PSF1 and PSF2.

Table S7. Adsorption capacity (q) and dye removal % of PSF1 and PSF2 toward single (MB) dye solution after recycling and desorption.

Adsorbent	PSF1 q(mg/g)	% dye removal PSF1	PSF2 q(mg/g)	% dye removal PSF2
**15 min after stir. filt.**	**7.62**	**59.7**	**11.9**	**93.5**
**1 st. cycle**	**7.62**	**59.7**	**11.9**	**93.5**
**2 nd. cycle**	**7.62**	**59.7**	**11.9**	**93.5**
**3 rd. cycle**	**7.62**	**59.7**	**11.9**	**93.5**
**4 th. cycle**	**0.022**	**29.4**	**0.0235**	**31.6**
